# Rapid genome‐wide evolution in *Brassica rapa* populations following drought revealed by sequencing of ancestral and descendant gene pools

**DOI:** 10.1111/mec.13615

**Published:** 2016-04-13

**Authors:** Steven J. Franks, Nolan C. Kane, Niamh B. O'Hara, Silas Tittes, Joshua S. Rest

**Affiliations:** ^1^ Department of Biological SciencesFordham University441 E. Fordham RoadBronxNY10458USA; ^2^ Department of Ecology and EvolutionThe University of Colorado at BoulderRamaley N122BoulderCO80309USA; ^3^ Department of Ecology and EvolutionStony Brook University650 Life SciencesStony BrookNY11794USA

**Keywords:** adaptation, *Brassica rapa*, climate change, contemporary evolution, natural selection, population genomics, rapid evolution

## Abstract

There is increasing evidence that evolution can occur rapidly in response to selection. Recent advances in sequencing suggest the possibility of documenting genetic changes as they occur in populations, thus uncovering the genetic basis of evolution, particularly if samples are available from both before and after selection. Here, we had a unique opportunity to directly assess genetic changes in natural populations following an evolutionary response to a fluctuation in climate. We analysed genome‐wide differences between ancestors and descendants of natural populations of *Brassica rapa* plants from two locations that rapidly evolved changes in multiple phenotypic traits, including flowering time, following a multiyear late‐season drought in California. These ancestor‐descendant comparisons revealed evolutionary shifts in allele frequencies in many genes. Some genes showing evolutionary shifts have functions related to drought stress and flowering time, consistent with an adaptive response to selection. Loci differentiated between ancestors and descendants (*F*_ST_ outliers) were generally different from those showing signatures of selection based on site frequency spectrum analysis (Tajima's *D*), indicating that the loci that evolved in response to the recent drought and those under historical selection were generally distinct. Very few genes showed similar evolutionary responses between two geographically distinct populations, suggesting independent genetic trajectories of evolution yielding parallel phenotypic changes. The results show that selection can result in rapid genome‐wide evolutionary shifts in allele frequencies in natural populations, and highlight the usefulness of combining resurrection experiments in natural populations with genomics for studying the genetic basis of adaptive evolution.

## Abstract 2

see also the Perspective by Hancock

## Introduction

With climate change and habitat loss threatening the viability of many species worldwide, understanding the ability of species to cope with these changes is crucial. Uncovering the genetic basis of adaptive evolution in natural populations contributes to this goal by aiding in assessment of the mechanisms and limits to adaptation (Franks & Hoffmann 2012). Several recent studies have made progress towards this goal by investigating adaptive genetic variation among populations over geographic space, including studies of local adaptation to serpentine soils in *Arabidopsis lyrata* (Turner *et al*. 2010), adaptation to variation in climatic conditions in *Arabidopsis thaliana* (Fournier‐Level *et al*. 2011; Hancock *et al*. 2011), variation in coat colour in natural populations of mice (Linnen *et al*. 2013) and repeated reductions of stickleback armour during adaptation to freshwater (Jones *et al*. 2012). A few studies have examined the genetic basis of very recent evolution, such as work showing altered frequencies of a chromosome inversion in *Drosophila subobscura* following climate change (Balanyá *et al*. 2006). However, we still know little about the genetic basis of contemporary adaptive evolutionary changes over time in natural populations (Rokas & Abbot 2009), in part because the genotypes of ancestral populations are typically unknown.

One solution to this problem is to combine genomics with resurrection experiments to study evolution. In resurrection experiments, ancestors and descendants are reared together from stored propagules and compared under common conditions (Franks *et al*. 2008). Differences between ancestors and descendants provide strong, direct evidence of evolutionary change. Studies using this approach have documented rapid evolutionary changes in phenotypic traits, such as flowering time (Franks *et al*. 2007; Nevo *et al*. 2012; Thomann *et al*. 2015). Combining resurrection experiments with genomics allows a direct assessment of the genetic basis of evolutionary change. This approach offers some distinct advantages over indirect methods of detecting signatures of selection such as those based on site frequency spectrum analyses, particularly in situations in which evolutionary changes are expected to result from selection acting on standing genetic variation (soft sweeps) rather than new or very low‐frequency mutations (hard sweeps). In addition, combining resurrection experiments with genomics is ideal for assessing the degree to which genetic responses to selection are parallel or divergent in different populations, which is a long‐standing question in evolution (Stern & Orgogozo 2009). Despite these advantages, genomics has previously only been combined with resurrection experiments in studies of laboratory populations, such as *Drosophila melanogaster* (Burke *et al*. 2010) and *Escherichia coli* (Blank *et al*. 2014).

Here we combine genomics with resurrection experiments using individuals from natural populations. The two natural populations of the annual plant *Brassica rapa* (field mustard) that are the subject of this study were previously shown to have undergone adaptive evolution in response to selection. These populations, located in southern California, experienced a series of wet years leading up to 1997 and then a series of dry years, in which drought was particularly severe late in the growing season, leading up to 2004 (Franks *et al*. 2007). A resurrection experiment compared ancestors obtained from seeds collected in 1997 to descendants derived from seeds collected in 2004. This experiment showed that the descendants flowered earlier than the ancestors in both populations, indicating rapid adaptive evolution that allowed the descendants to escape the late‐season drought (Franks *et al*. 2007; Franks 2011). Further work on these populations showed evolutionary shifts to more rapid development (Franks & Weis 2008) and increased susceptibility to pathogenic disease (O'Hara *et al*. 2016). This prior work thus demonstrated rapid evolutionary changes in multiple phenotypic traits, but the underlying genetic basis of these evolutionary shifts remained unknown.

In this study, we investigated the genetic basis of these documented contemporary evolutionary changes through direct comparisons of predrought (1997) ancestral and postdrought (2004) descendant gene pools in two southern California populations of *B. rapa*. We assessed evolutionary changes in allele frequencies by sequencing the genomes of 205 plants derived from ancestral and descendant seeds.

## Materials and methods

### Study system

*Brassica rapa* L. (Brassicaceae), commonly known as field mustard, is an annual, obligate outcrossing, herbaceous plant introduced into the United States. Although considered a mesopolyploid, it behaves functionally as a diploid, and has been fully sequenced (Wang *et al*. 2011). We studied *B. rapa* populations in southern California. *B. rapa* plants in this location germinate and begin to grow with the first rains, which generally occur around December or January, and continue to grow and produce flowers as long as conditions remain favourable, which can last from several weeks to several months (Franks *et al*. 2007). As in previous studies (Franks *et al*. 2007; Franks & Weis 2008, 2009; Franks 2011), we sampled two populations: Arboretum (Arb) and Back Bay (BB). The BB site has soils that are more well drained than the Arb site, and the two sites are about 3 km apart. Both populations evolved earlier flowering time in response to the drought, with the evolutionary response greater in the Arb population (Franks *et al*. 2007). In both populations, the drought had the effect of shortening the growing season, which favoured earlier flowering plants, but did not appear to cause a strong population bottleneck, as we did not observe any noticeable declines in population size. The populations contained thousands to tens of thousands of individuals.

### Plant propagation

We collected seeds from thousands of individuals from each population before (1997) and after (2004) the drought. From each of these collections, 500 seeds were randomly selected and grown under greenhouse conditions in 2006. Based on the large number of plants that served as the source of the collection, and the large number of seeds used, we do not expect that this procedure would have caused a strong population bottleneck. Plants were crossed randomly within each group (population and generation), and seeds from these intrapopulation crosses were collected to generate ancestral and descendant lines (Franks *et al*. 2007). At the start of the current study in 2012, seeds from ancestral and descendant lines were planted in pots containing growing medium (Sunshine Mix #1; Sungro) on light carts under constant (24 h/day) light, and watered daily. In both greenhouse generations, germination rates were high (>90%) in all groups and there were no differences in germination rates between groups (*P* > 0.05), ensuring the plants represent an unbiased sample of the gene pools of the original populations. Our set of samples subjected to sequencing contained 205 accessions derived from the predrought (1997) and postdrought (2004) seed samples from both the Arb (50 pre‐ and 74 postdrought) and BB populations (22 pre‐ and 59 postdrought).

### Sequencing

True (noncotyledon) leaves collected after 10–14 days of growth were lysed (FastPrep; MP Bio) followed by genomic DNA extraction (DNeasy Plant Mini Kit; Qiagen). We then normalized DNA concentrations among samples because we wanted to make sure that each individual contributed equally to the pooled sample for each library. We normalized using quantitative PCR of the GAPDH gene, which is commonly used in qPCR studies. This gene was chosen because it is a housekeeping gene with important function, and thus is unlikely to display polymorphism (Wedel & Soll 1998), and it has stable expression even under drought conditions (Qi *et al*. 2010), so should be unaffected by the drought conditions experienced by the plants in our natural populations. Our primers for the GAPDH gene were forward (5′–3′): gaaaggtgcttccacagctc and reverse (5′–3′): gtcgcagctttctcgagtct (Untergasser *et al*. 2007). Samples were run in duplicate using Power SYBR Green on a 7300 Real‐Time PCR System (Applied Biosystems) and conditions: 50 °C (10 min), 95 °C (5 min), 40 × [95 °C (30 s), 55 °C (1 min) 72 °C (30 s)], 95 °C (15 s), 55 °C (30 s) and 95 °C (15 s). Equimolar amounts of DNA were then pooled within each population and year, with two technical replicates of each pool. Each pool was used to construct a Nextera (Illumina) library and sequenced on three lanes of Illumina HiSeq.

### Read processing and population genomics analysis

The reads were paired end and were 100 bp long. There were on average 9 million high‐quality reads per pool, and approximately 70% of these could be aligned to the reference genome and mapped. We ran a custom script to remove the first nine bases, which had low quality scores, trim the ends of reads with a quality score of <20, filter low complexity regions, remove nucleotides with 0 quality and remove library barcodes. We used the aln algorithm in BWA (Li & Durbin 2009) to align pooled population sequences to the *B. rapa* reference genome (version 1.18) (http://brassicadb.org/brad/). Default parameters were used with the exception that *q* (quality threshold for read trimming to 35 base pairs) was changed from 0 to 20 to reduce read trimming (in addition to read trimming performed above). We used samtools (Li *et al*. 2009) to generate a separate multi pileup file for each population.

Population genetic statistics (*F*_ST_ and Tajima's *D*) were calculated using the packages popoolation (Pandey *et al*. 2011) and popoolation2 (Kofler *et al*. 2011), with default parameters except as indicated in the Supporting information. There was a high degree of concordance in population genetic parameter estimates between the two technical replicates, indicating that these results are robust to technical differences in pooling or sequencing (see Supporting information). Therefore, analysis presented here was performed on data combining both replicates of each population.

We conducted separate genewise and windowed *F*_ST_ analyses. The genewise *F*_ST_ analyses were conducted over the entire coding region of the gene, making sure that each gene included had at least four SNPs. The windowed *F*_ST_ analyses were conducted in 100‐kb windows across the genome. Statistical significance was determined for genewise *F*_ST_ by comparison of the test distribution to a null distribution. The null distribution was created with 1000 replicates of bootstrap resampling in r version 3.1.2 (R Core Team 2014). We then regressed the test distribution on the null distribution and used the outlierTest function in the car package (Fox & Weisberg 2011) in r to calculate *P*‐values for the residuals in the regression. We corrected for multiple statistical tests using false discovery rate (FDR)‐corrected *q*‐values with fdrtool (Blank *et al*. 2014). We set the significance threshold at FDR *q*‐values of <0.05 (Storey & Tibshirani 2003), similar to a cut‐off of a *P*‐value of *P* < 0.05, and indicating an expected proportion of false discoveries of 5%. All genes included in the analysis had a minimum of four SNPs.

### Validation of pooled allele frequencies

To confirm our pooled results at the individual level, we performed Kompetitive Allele‐Specific PCR genotyping (KASP; LGC Genomics). We chose 10 SNPs from well‐annotated loci with an *F*_ST_ value >0.2 in at least one population (Table S1, Supporting information). We tested 29 samples from each population (BB and Arb) for each time period (ancestors and descendants) for a total of 116 samples. These 116 samples were chosen randomly from among those for which we had sufficient quantities of DNA.

Nine of our ten chosen SNPs amplified consistently and were used for the validation analysis. We found high concordance in allele frequencies obtained from KASP genotyping compared to pooled Illumina sequencing (Pearson correlation *r* = 0.83, *P* < 0.001; Fig. S1, Supporting information), indicating that our next‐generation sequencing of pooled DNA from multiple individuals in each population provides equivalent estimates of allele frequencies in those populations to estimates that are based on individual sample genotyping.

### Gene ontology analysis

Gene ontology (GO) analysis was performed using ErmineJ, with annotations from *Arabidopsis thaliana* (Gillis *et al*. 2010). As *F*_ST_ distributions were non‐normal, we conducted a receiver operator characteristic analysis, which uses ranked gene scores. Significance was calculated using previously described algorithms (Breslin *et al*. 2004) and *P*‐values were corrected using a FDR correction.

## Results

Our low‐coverage (~25× per pooled library) whole‐genome shotgun sequencing identified 5 812 602 sites with SNPs. Within regions annotated as coding sequence, we found 42 360 high‐quality, biallelic SNPs segregating at moderate to high frequency (a minor allele frequency of more than 5%) in these populations. Average overall nucleotide diversity per site (π) was 0.00957.

**Figure 1:**
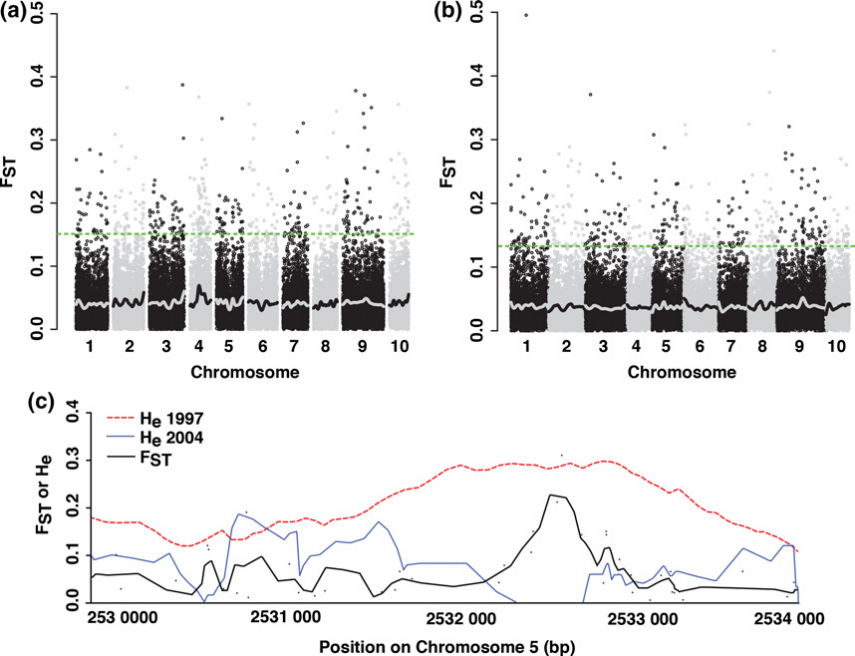
Genetic differentiation (*F*_ST_) throughout the genome between predrought ancestors (1997) and postdrought descendants (2004) of *Brassica rapa* in the (a) Arboretum and (b) Back Bay populations, and (c) within one flowering time gene in the Arb population. For (a) and (b), each point is a gene, and average *F*_ST_ was calculated for each gene using 100‐kb sliding windows. The green dashed line indicates the significance cut‐off (*q*‐value < 0.05), of *F*_ST_ > 0.15 for Arb and *F*_ST_ > 0.13 for BB. The number of significantly differentiated genes was 434 for Arb and 433 for BB. This shows evidence for rapid evolutionary shifts in these genes. A LOESS trend line is shown in black and grey. In (c), differentiation (*F*_ST_) between ancestors and descendants and expected heterozygosity (*H*_e_) from 1997 (red dashed line) and 2004 (blue dotted line) Arb populations are shown for 4 kb of Bra004928, the *SOC1* paralog on chromosome 5. This region shows high *F*_ST_ and a decrease in *H*_e_ from 1997 to 2004, providing potential evidence of recent selection.

To identify genes showing evolutionary shifts in the Arb and BB populations, we conducted outlier fixation index (*F*_ST_) analysis (Lewontin & Krakauer 1973) comparing ancestors and descendants within each population. Here, genes with outlier *F*_ST_ values differ in allele frequencies between ancestors and descendants and thus evolved between 1997 and 2004. We found that many genes throughout the genome were significantly differentiated (had outlier *F*_ST_ values) between ancestors and descendants in both populations (Fig. 1a–b; Data S1, Supporting information). We found 855 genes (~2% of genes in the analysis) that were outliers in one or both populations (*F*_ST_ > 0.15 in Arb, *F*_ST_ > 0.13 in BB; after false discovery correction). Thus, during the course of the drought there were rapid changes in allele frequencies in multiple genes in both populations. The average genome‐wide (genic and nongenic) between‐year *F*_ST_ was 0.035 (0.034–0.035) for Arb and 0.032 (0.031–0.033) for BB (95% bootstrapped CIs in parentheses). These values, while very low, were significantly greater than zero, suggesting that rapid evolutionary shifts occurred at both coding and noncoding loci. For comparison, genome‐wide *F*_ST_ between the Arb and BB populations (which are separated over space rather than over time) was 0.044 (0.043–0.045) in 1997 and 0.047 (0.045–0.048) in 2004.

**Figure 2:**
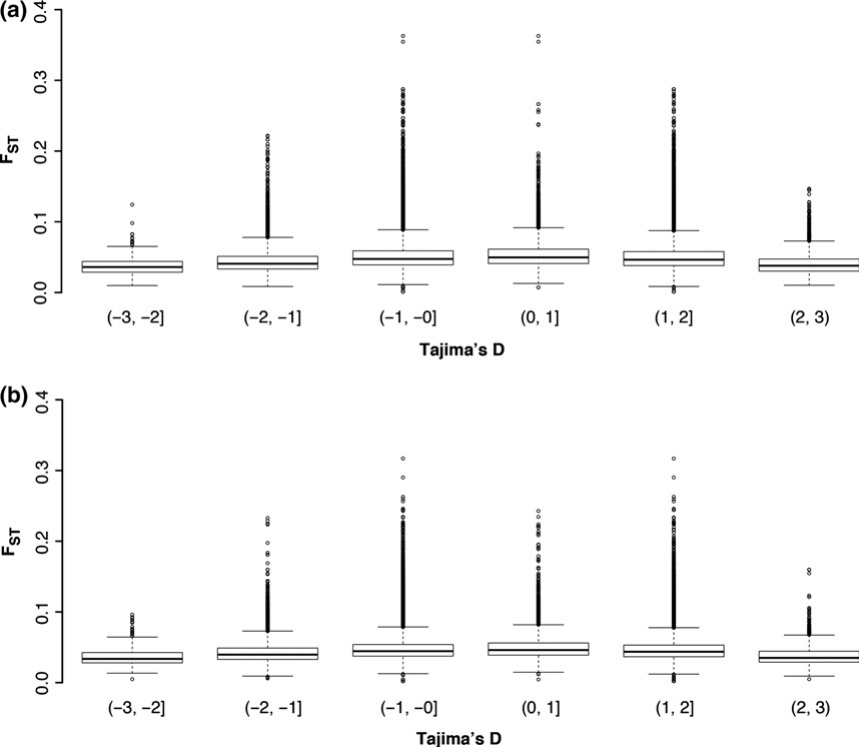
Boxplots of *F*_ST_ at different ranges of Tajima's *D* for (a) Arb and (b) BB descendants (2004). There is no apparent correlation between Tajima's *D* and *F*_ST_, indicating that regions that were differentiated between 1997 and 2004 (high *F*_ST_) are not the same as regions that were under longer‐term selection (low Tajima's *D*). Instead, genes with extreme Tajima's *D* values tended to have low *F*_ST_ values.

We used the site frequency spectrum test Tajima's *D* (Simonsen *et al*. 1995) to detect potential historical selection. Multiple loci showed extreme (<−2 or >2) Tajima's *D* values in both ancestors and descendants (Fig. S2, Supporting information). Tajima's *D* values were strongly correlated before and after the drought for both Arb (Pearson correlation *r* = 0.77, *P* < 0.0001) and BB (*r* = 0.79, *P* < 0.0001), indicating a lack of a genome‐wide effect of this recent evolutionary event on Tajima's *D*. In addition, the loci identified by Tajima's *D* and outlier *F*_ST_ analyses tended to be distinct (Fig. 2), indicating likely differences between the genes under historical selection and those involved in responses to the recent drought.

Of the 855 ancestor‐descendant *F*_ST_ outlier genes, 85% have identified homologues in *Arabidopsis*, based on the *Brassica* genome database (http://brassicadb.org/brad/). Many of the outlier genes we detected have annotations related to drought response traits (Table 1; Data S1, Supporting information). For example, one of top *F*_ST_ outlier genes, Bra004928, is one of three *B. rapa* homologues to *SOC1*, which is known to play a central role in regulating flowering time (Immink *et al*. 2012). This gene showed strong reductions in genetic variation in both Arb and BB populations after the drought, with reduced expected heterozygosity (*H*_e_) in descendants as compared to ancestors (shown for Arb in Fig. 1c). The reductions in variation (*H*_e_) and high divergence (*F*_ST_) are focused on a relatively narrow region within *SOC1*, with both 2004 populations showing an elimination of variation (*H*_e_ = 0) at many SNPs. This result suggests strong recent selection acted on variation within this gene. Other outlier genes are related to flowering time and hormones that influence flowering time, such as gibberellin, as well as drought and osmotic stress tolerance (Table 1; Data S1, Supporting information).

**Table 1:** Genes that evolved during the course of a drought, as indicated by outlier *F*_ST_ values

Brassica gene	Chr.	Pos. (Mb)	*F*_ST_ Arb	*F*_ST_ BB	Arabidopsis homologue	Gene name	Gene annotation
Bra028847	2	1.8	**0.26**	0.05	AT5G03180		RING/U‐box superfamily protein
Bra008462	2	15.8	**0.22**	**0.29**	AT2G26000	BRIZ2	Zinc finger (C3HC4‐type) protein
Bra020688	2	23.8	0.03	**0.26**	AT5G13930	CHS	Chalcone and stilbene synthase protein
Bra023070	3	8.5	0.02	**0.24**	AT2G36450	HRD	Integrase‐type DNA‐binding protein
Bra000731	3	12.7	**0.17**	**0.24**	AT4G11040		Protein phosphatase 2C family protein
Bra004928	5	2.5	0.13	**0.24**	AT2G45660	SOC1	AGAMOUS‐like 20
Bra018560	5	9.3	0.04	**0.29**	AT4G02270	RHS13	Root hair‐specific 13
Bra022378	5	18.1	0.04	**0.23**	AT3G19010		2‐Oxoglutarate (2OG) and Fe(II)‐dependent oxygenase protein
Bra040610	5	23.5	**0.25**	0.06	AT3G02910		AIG2‐like (avirulence) protein
Bra018989	6	0.9	0.04	**0.32**	AT1G52790		2‐Oxoglutarate (2OG) and Fe(II)‐dependent oxygenase protein
Bra019917	6	3.7	**0.25**	**0.19**	AT2G39540		Gibberellin‐regulated protein
Bra019637	6	5.4	**0.25**	0.04	AT3G52960		Thioredoxin protein
Bra026216	6	5.5	**0.32**	0.10	AT1G14470		Pentatricopeptide repeat protein
Bra016370	8	18.1	**0.31**	0.07	AT1G79680	WAKL10	WALL ASSOCIATED KINASE
Bra016605	8	19.3	0.03	**0.44**	AT1G78610	MSL6	Mechanosensitive channel of small conductance‐like 6
Bra030650	8	20.9	**0.35**	0.02	AT1G06360		Fatty acid desaturase protein
Bra035879	9	3.2	**0.24**	0.06	AT5G62850	AtVEX1	Nodulin MtN3 protein
Bra037300	9	4.5	**0.29**	0.12	AT4G33270	CDC20.1	Transducin protein/WD‐40 protein
Bra023306	9	19.7	**0.32**	**0.17**	AT3G27890	NQR	NADPH:quinone oxidoreductase
Bra007772	9	30.8	0.10	**0.23**	AT2G26040	PYL2	PYR1‐like 2

The top 20 most divergent well‐annotated genes, including *Arabidopsis* best hits (Cheng *et al*. 2011) and *F*_ST_ values calculated between ancestors and descendants for the Arb and BB populations of *Brassica rapa*. *F*_ST_ values significant at a *q*‐value of 0.05 are indicated with bold. For the full list of all outlier genes, see Data S1 (Supporting information).

We examined the functional annotations of differentiated genes using gene ontology analysis weighted by *F*_ST_ values, and found significant enrichment of genes associated with circadian rhythm (GO 0042752, *P* = 0.02) and protein phosphatase type 1 (GO 0000164, *P* = 0.05) in the Arb population [which showed a much greater shift in flowering time than the BB population (Franks *et al*. 2007)], and no significant enrichment in the BB population.

**Figure 3:**
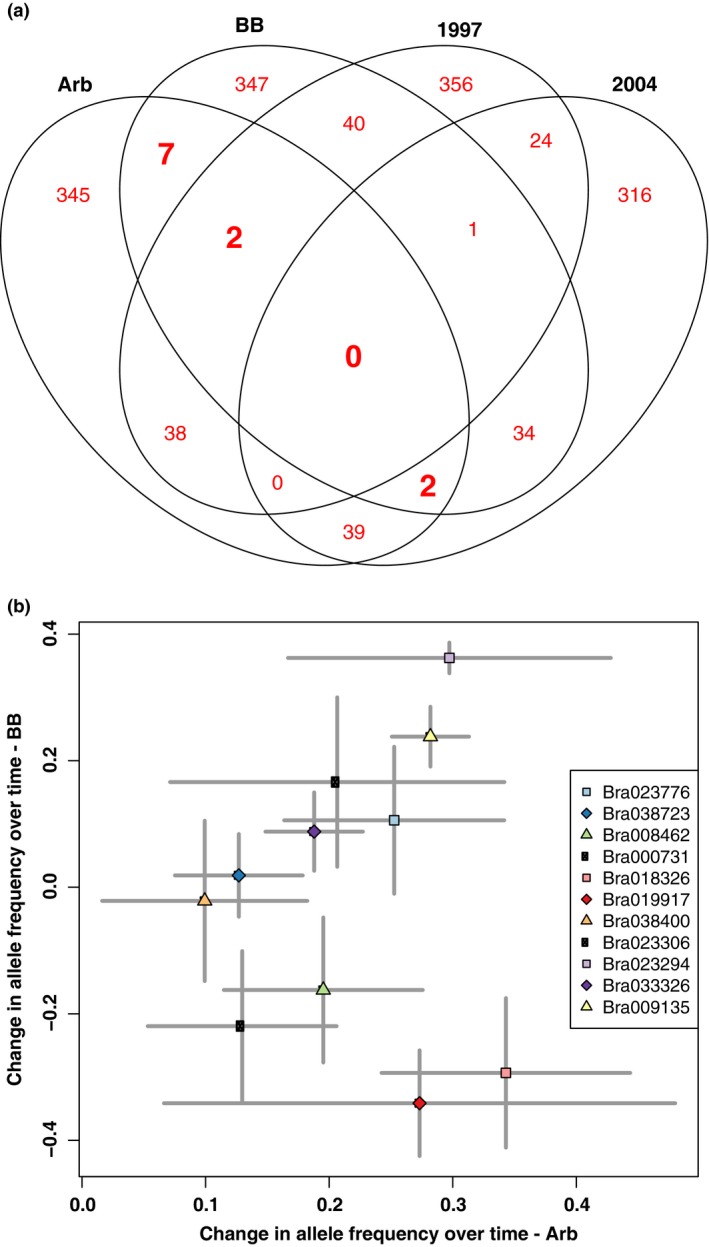
Few genes evolved in parallel in the Arb and BB populations. (a) Venn diagram of the outlier genes significantly differentiated (*q*‐value < 0.05) between all temporal and geographic populations. For example, the numbers in the ellipse labelled Arb are the number of outlier genes that were outliers between ancestors and descendants in the Arb population. The genes that overlap between the Arb and BB ellipses (11 genes, shown in bold) are the genes that were outliers in both the Arb and BB populations. (b) Comparison of changes in ancestor‐descendant allele frequencies in Arb and BB populations for the 11 genes with significant differentiation in both populations. Points are coloured by gene (see legend). The major allele for Arb in 2004 is always tracked for each locus. The central point is the mean, and bars represent standard errors. The lack of correlated change between the two populations indicates little evidence for parallel evolution.

Of the genes significantly differentiated between ancestors and descendants in Arb (434 genes) and BB (433 genes), only 11 (0.025%) are found in both populations (compared to a null expectation of 5.6, *P* < 0.0001, exact binomial test; Fig. 3a; Table S1, Supporting information). Allele frequencies did not shift in parallel between the two populations for these 11 genes more than expected by chance (Pearson correlation *r* = 0.03, *P* = 0.85; Fig. 3b).

## Discussion

Our results clearly indicate a genetically based, rapid evolutionary change in both natural populations over the 6 years of the drought period. The previously demonstrated phenotypic shifts shown under common conditions (Franks *et al*. 2008) are here corroborated by allele frequency differences between ancestors and descendants throughout the genome. Our resurrection genomics approach allowed us to observe genetic changes in the populations directly, uncovering clear evidence of the evolution of allele frequencies, which likely include the alleles responsible for the evolutionary change in phenotypes. To determine which of the loci showed significant shifts and to account for the potential of many false positives, we used an outlier *F*_ST_ approach coupled with a *q*‐value threshold correction. Although the outlier *F*_ST_ approach has several limitations for inferring past selection from contemporary populations (Narum & Hess 2011; Bierne *et al*. 2013; De Mita *et al*. 2013), we avoid many of these issues in our study by comparing recent ancestors and descendants directly. This approach indicated that the gene pools of ancestors and descendants in both populations showed small but distinct differences in allele frequencies, with *F*_ST_ significantly greater than zero, based on a large number of loci in coding and noncoding regions throughout the genome. This result is comparable to that of a previous study on these populations using microsatellite markers (Welt *et al*. 2015). It thus appears that genetic changes occurred in these populations over a very short period of time, supporting the idea that evolutionary changes in natural populations can occur rapidly enough to be observed. In all cases, we observed shifts in the frequency of alleles already present in the ancestors, consistent with selection acting on standing genetic variation (soft sweeps) rather than new or very low‐frequency mutations (hard sweeps), as expected given the short period of time, and as found in previous studies of laboratory populations using a similar approach (Burke *et al*. 2010).

In addition to direct comparisons of ancestors and descendants, we also used site frequency spectrum analyses to look for signatures of past selection. We found that genes under long‐term or historical selection in these populations are generally distinct from the genes that responded to the most recent selective episode caused by the drought. This conclusion is based on comparing *F*_ST_ outliers to genes identified as under selection by site frequency spectrum analysis. We found multiple loci showing extreme values of Tajima's *D*. However, Tajima's *D* values were strongly correlated before and after the drought, indicating a lack of effect of this recent selective event on Tajima's *D*. Furthermore, the loci detected by Tajima's *D* and by outlier *F*_ST_ analyses were distinct. This indicates that Tajima's *D* and outlier *F*_ST_ analyses detected selection acting at different temporal scales. This result is predicted by theory (Simonsen *et al*. 1995; Biswas & Akey 2006), but the results here provide empirical support. The approach of combining resurrection experiments with genome‐wide sequencing that we used here may thus have important advantages for investigating the genetic basis of very recent, rapid evolution. The distinctness of genes under historical selection, as indicated by Tajima's *D*, and recent selection, as indicated by *F*_ST_, suggest that some elements of the climatic change experienced during this drought may have been different between 1997 and 2004 than in previous events that shaped standing genetic population structure. However, the expected degree of similarity between short‐term compared to long‐term evolutionary genetic signatures is generally not known, and would be a useful area of enquiry.

Because our study included two populations that both experienced the drought, with both showing phenotypic evolutionary changes in the same direction, we could also examine the degree of concordance in genetic changes in these populations. The level of genetic differentiation between the populations before the drought was low but significant, indicating overall similarity but some differences in their starting condition. We found that genomic responses to the same drought event in two geographic populations with similar phenotypic responses had distinct genetic bases. Genetic changes in the Arb and BB populations were mainly independent rather than parallel. Of the genes that shifted in allele frequency, only 0.025% were in common between the two populations. Although this is greater than expected by chance, the proportion of overlapping loci is still far smaller than expected if the genetic basis of the evolutionary changes were parallel in the two populations. Furthermore, for the 11 genes that were differentiated in both populations, there was no trend indicating that the shifts tended to be of the same magnitude and direction. This independent trajectory of the genetic basis of evolution in the two populations could be due to the fact that although both experienced drought, the effects and phenotypic responses were stronger in the Arb than in the BB population (Franks *et al*. 2007). Alternatively, it could also be that shifts in different loci can achieve similar phenotypic results, providing different genetic solutions to the same adaptive problem. Indeed, the general question of how often and under what conditions the genetic basis of evolutionary changes in different populations is expected to be parallel or divergent, and to what extent genetic changes are predictable, is still very much open (Stern & Orgogozo 2009). Future experimental evolution studies with multiple descendant populations under controlled conditions may be a useful approach to addressing this question.

A key question in evolutionary biology is to what extent shifts in allele frequencies are due to selection or other evolutionary forces such as genetic drift. Our study showed rapid changes in allele frequencies in two populations, which demonstrates evolution, but this result alone does not indicate whether genetic changes we observed were due to selection or drift. However, there are several lines of evidence suggesting that selection played at least some role in the genetic changes observed. First, we know from our previous work that selection acted on these populations during the drought and that the resulting phenotypic evolutionary changes were adaptive (Franks *et al*. 2007). This does not mean that selection acted on all differentiated loci or that all genetic changes we observed represent adaptation, but it does indicate that selection and adaptation occurred and could have contributed to changes in allele frequencies. Second, some of the loci most differentiated between ancestors and descendants include genes with annotations consistent with expected responses to drought, including flowering time, circadian rhythm and stress response. In particular, one homologue of *SOC1*, a gene known to be a centrally important regulator of flowering time (Immink *et al*. 2012), was found to be highly differentiated in both populations, and to have reductions in genetic diversity consistent with recent response to selection. Although individual candidate loci would need to be confirmed with further experimental work, ideally involving genetic manipulations and fitness tests (Pardo‐Diaz *et al*. 2015), the annotations of these genes provides compelling evidence for the involvement of selection in at least some of the evolutionary changes observed. Third, it is unlikely that the genetic changes we observed were the effect of a genetic bottleneck. The drought that occurred in these populations was a late‐season drought that favoured early‐flowering plants (Franks *et al*. 2007) but did not appear to result in any mass mortality event or decline in populations size, based on our observations of these populations. Furthermore, there were no major reductions in genetic diversity, losses of alleles or declines in heterozygosity in the ancestors of either population that would have been consistent with a genetic bottleneck. Thus, it did not appear that the drought caused a bottleneck. Despite the lack of a bottleneck, it is still possible that allele frequencies at some loci shifted due to drift. However, it is unlikely that drift would have caused such a substantial change in allele frequency at many loci in large populations in just a few generations. Although the general lack of parallel genetic changes could indicate drift, there were some genes associated with phenotypes of interest that did evolve in both populations in parallel, and the divergence could also indicate that both populations evolved in response to selection but showed largely independent changes. Taken together, this evidence strongly suggests that selection played a role in at least some of the evolutionary genetic changes observed, and not all of the changes were purely due to drift alone.

In summary, the results of this study demonstrate rapid genetic changes in populations that evolved in response to drought. This work provides novel insights into the genetic mechanisms of evolution in response to drought in these populations. The research also illustrates the value of combining genomics with the resurrection approach to studying evolution.

## Data accessibility

All sequences have been deposited in the Short Read Archive: PRJNA277879: Rapid genome‐wide evolution in *Brassica rapa* populations following drought revealed by sequencing of ancestral and descendant gene pools (TaxId: 3711) with 4 biosamples: SAMN03397805: Back Bay 1997 (TaxId: 3711); SAMN03397806: Back Bay 2004 (TaxId: 3711); SAMN03397807: Arboretum 1997 (TaxId: 3711); and SAMN03397808: Arboretum 2004 (TaxId: 3711).

N.B.O. conducted the laboratory work; N.C.K., J.S.R. and S.J.F. designed the experiments; all authors contributed to writing and editing the manuscript; and all authors contributed to data analyses.

## Supporting information


**Fig. S1.** High correlation between KASP and Illumina next‐generation determined allele frequencies in *Brassica rapa* populations.

**Fig. S2.** Tajima's *D*, calculated using a 100‐kb sliding window, shown across the genome for ancestral (blue) and descendant (red) populations with trend lines added using a local regression smoothing with a span of 0.05.

**Table S1.** SNPs chosen for KASP validation of *Brassica rapa* samples.

**Table S2.** Genes showing evolutionary shifts in both populations.

**Data S1.** All *F*_ST_ outlier genes, which evolved during the course of a drought.

**Data S2.** Methods.
