# Supplementary figures and images for: Rapid genome‐wide evolution in Brassica rapa populations following drought revealed by sequencing of ancestral and descendant gene pools
Source: Mol Ecol. 2016 Apr 13;25(15):3622–31. doi: 10.1111/mec.13615 (PMC4963267; doi:10.1111/mec.13615)

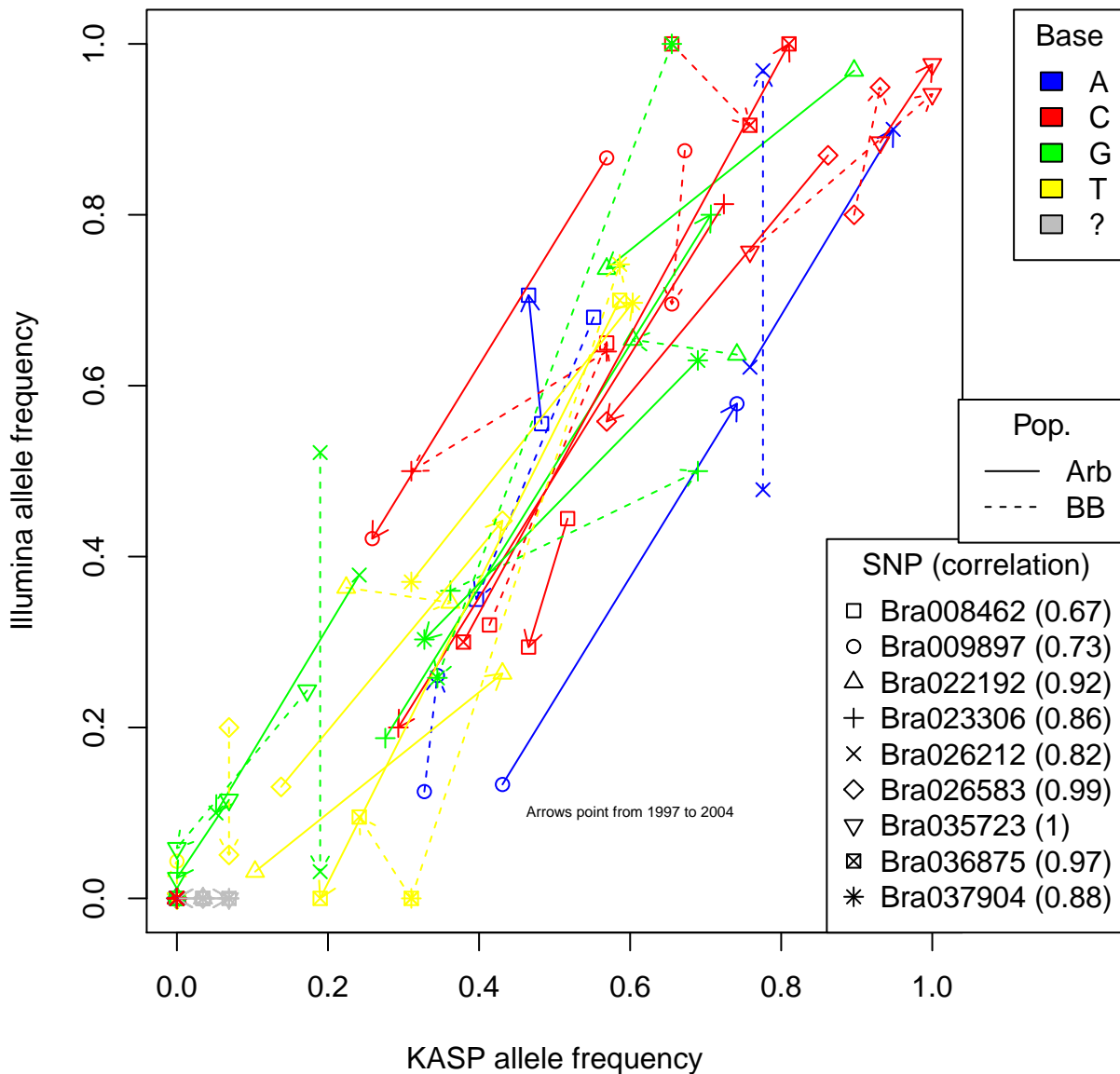

Supplement: Supplementary file 1 — Fig. S1. High correlation between KASP and Illumina next‐generation determined allele frequencies in Brassica rapa populations. [file MEC-25-3622-s001.pdf]
