# Supplementary material for: Rapid genome‐wide evolution in Brassica rapa populations following drought revealed by sequencing of ancestral and descendant gene pools
Source: Mol Ecol. 2016 Apr 13;25(15):3622–31. doi: 10.1111/mec.13615 (PMC4963267; doi:10.1111/mec.13615)
Supplement: Supplementary file 2 — Fig. S2. Tajima's D, calculated using a 100‐kb sliding window, shown across the genome for ancestral (blue) and descendant (red) populations with trend lines added using a local regression smoothing with a span of 0.05. [file MEC-25-3622-s002.pdf]

Fig. S2

A

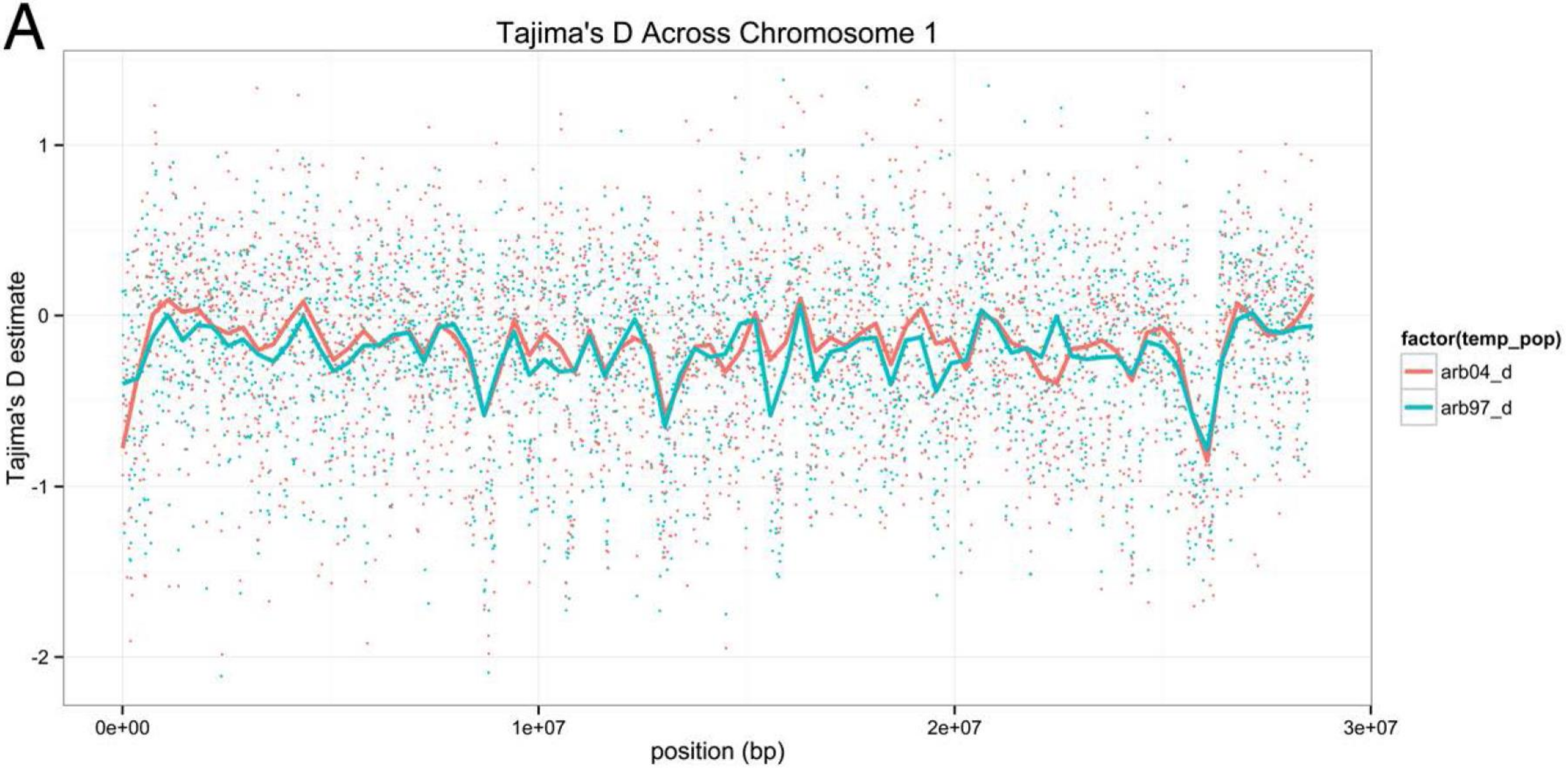

Tajima's D Across Chromosome 1
Tajima's D estimate
1
0
-1
-2
0e+00
1e+07
2e+07
3e+07
position (bp)
factor(temp_pop)
arb04_d
arb97_d

B

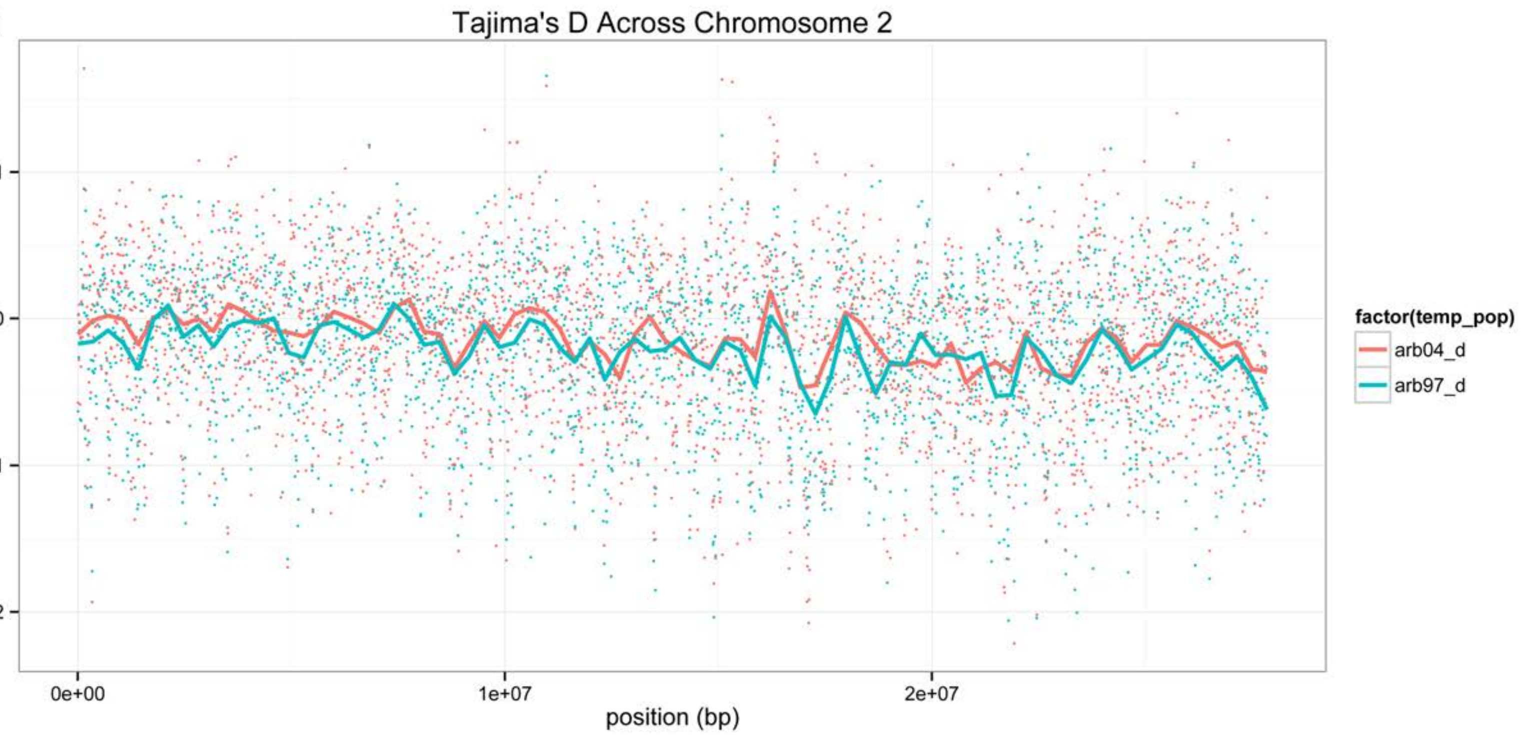
Tajima's D Across Chromosome 2
Tajima's D estimate
1
0
-1
-2
0e+00
1e+07
2e+07
position (bp)
factor(temp_pop)
arb04_d
arb97_d

C

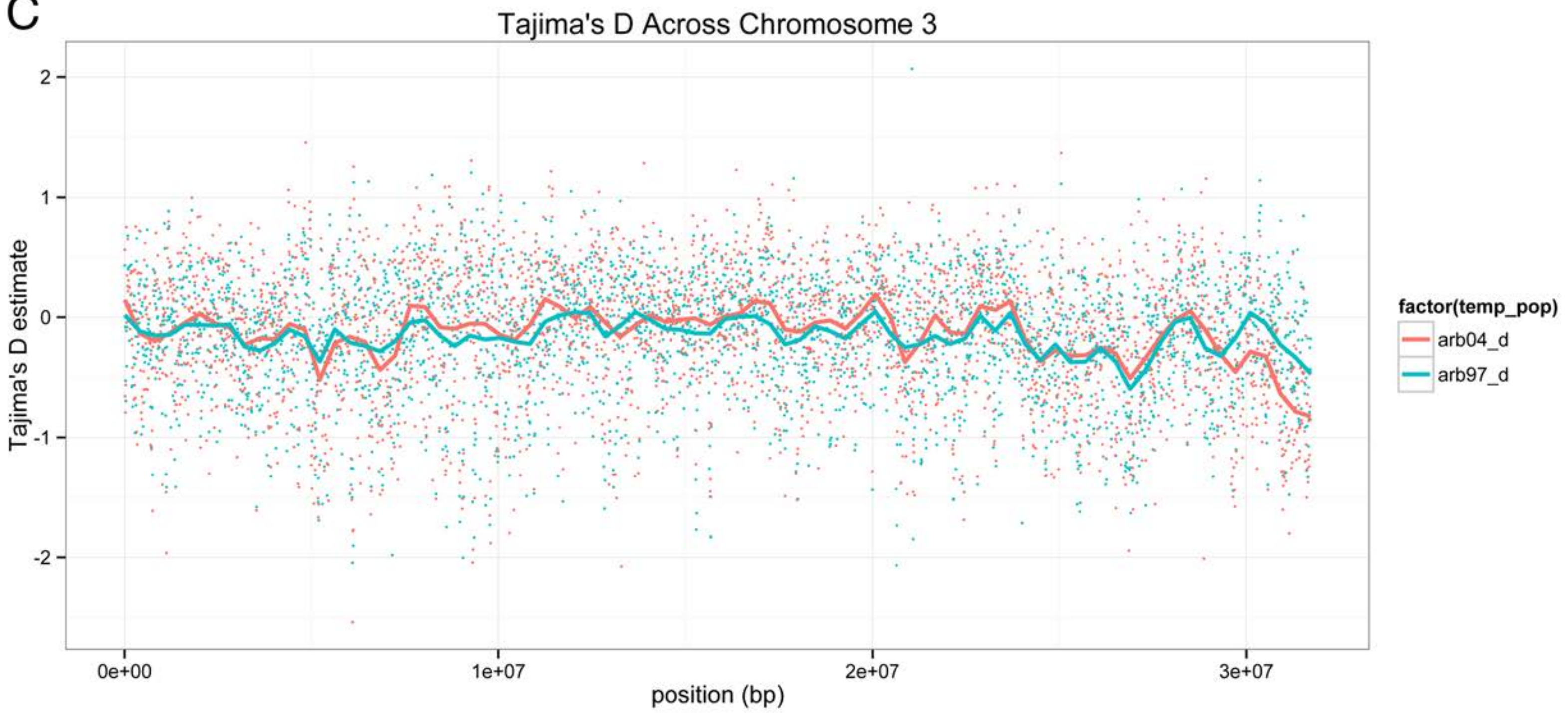
Tajima's D Across Chromosome 3
Tajima's D estimate
2
1
0
-1
-2
0e+00
1e+07
2e+07
3e+07
position (bp)
factor(temp_pop)
arb04_d
arb97_d

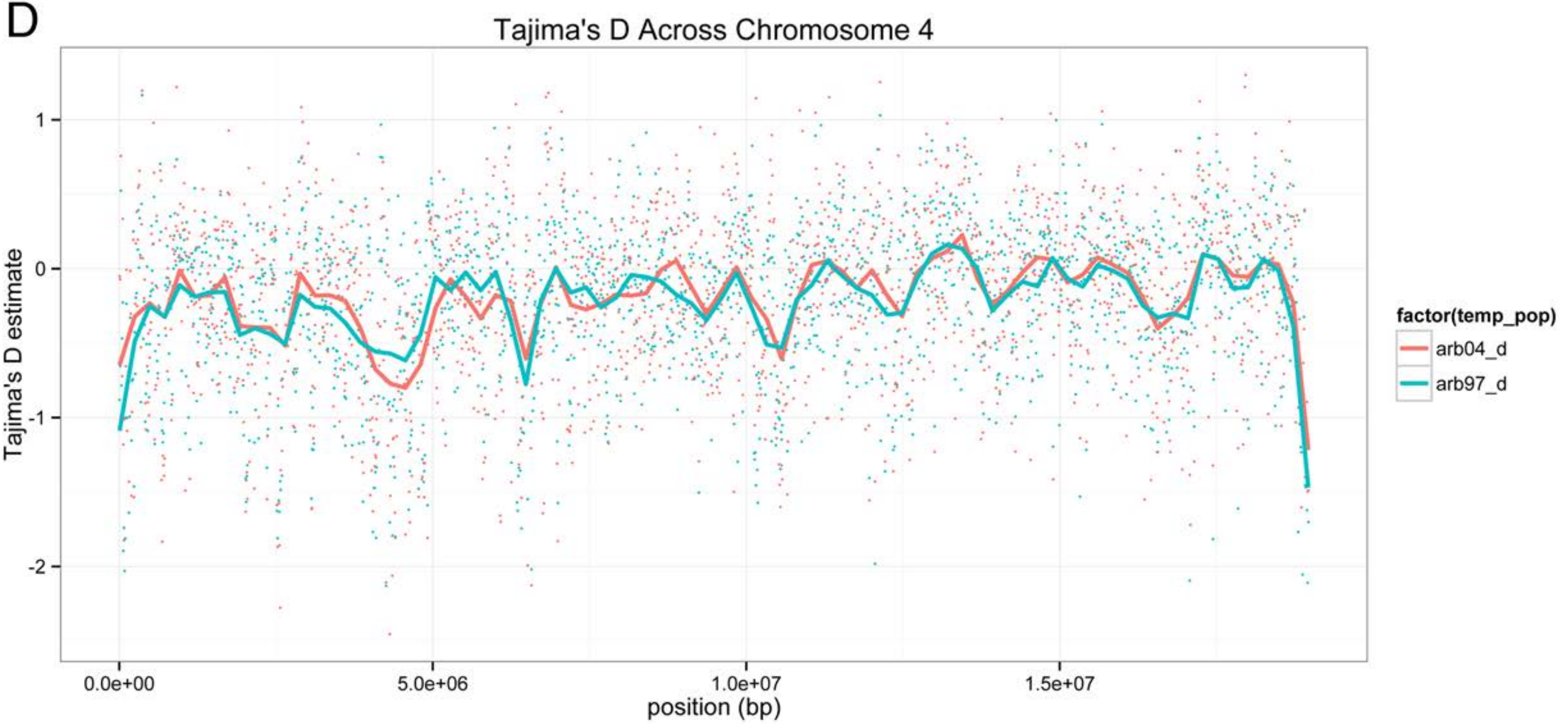

D
Tajima's D Across Chromosome 4
Tajima's D estimate
1
0
-1
-2
0.0e+00
5.0e+06
1.0e+07
1.5e+07
position (bp)
factor(temp_pop)
arb04_d
arb97_d

E

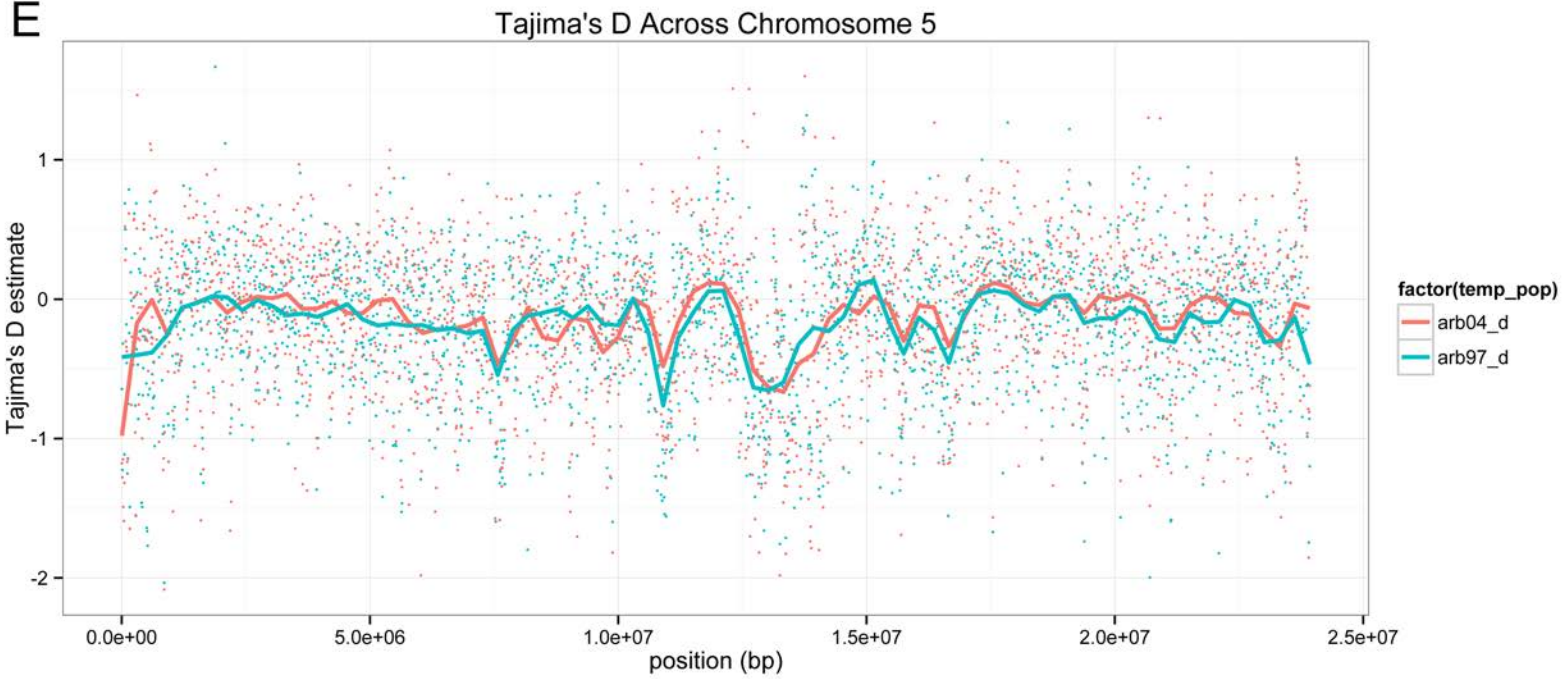
Tajima's D Across Chromosome 5
Tajima's D estimate
1
0
-1
-2
0.0e+00
5.0e+06
1.0e+07
1.5e+07
2.0e+07
2.5e+07
position (bp)
factor(temp_pop)
arb04_d
arb97_d

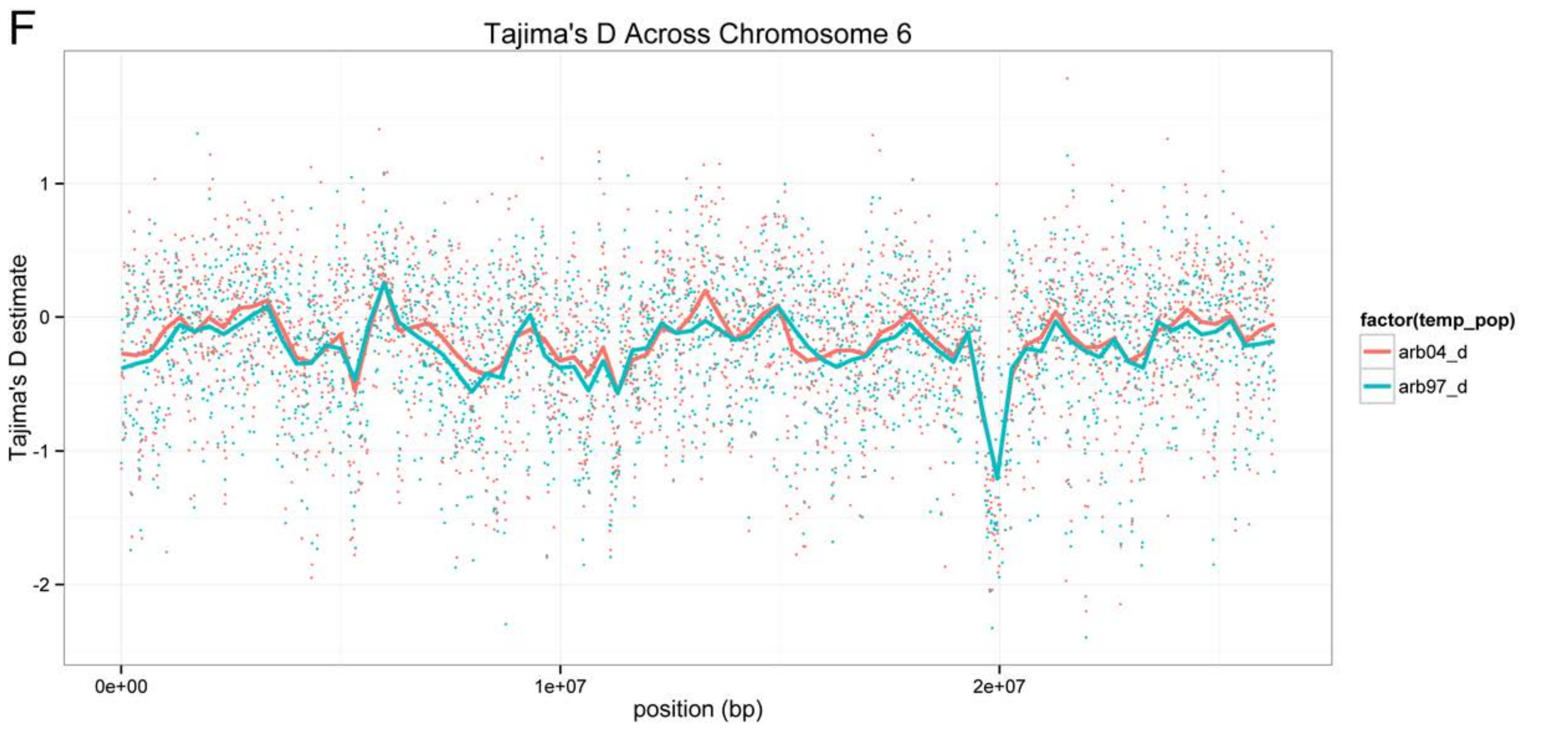
F
Tajima's D Across Chromosome 6
Tajima's D estimate
1
0
-1
-2
0e+00
1e+07
2e+07
position (bp)
factor(temp_pop)
arb04_d
arb97_d

G

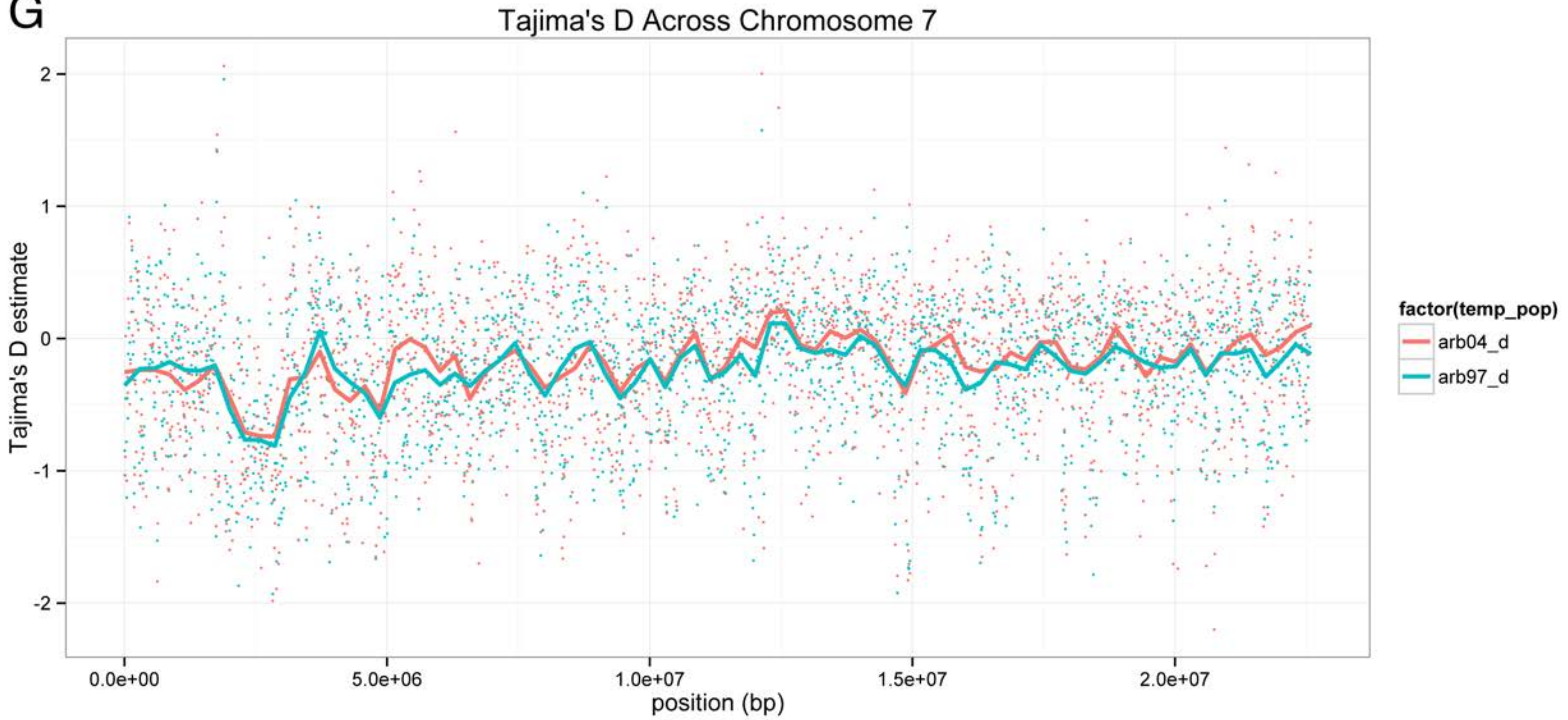

Tajima's D Across Chromosome 7
Tajima's D estimate
2
1
0
-1
-2
0.0e+00
5.0e+06
1.0e+07
1.5e+07
2.0e+07
position (bp)
factor(temp_pop)
arb04_d
arb97_d

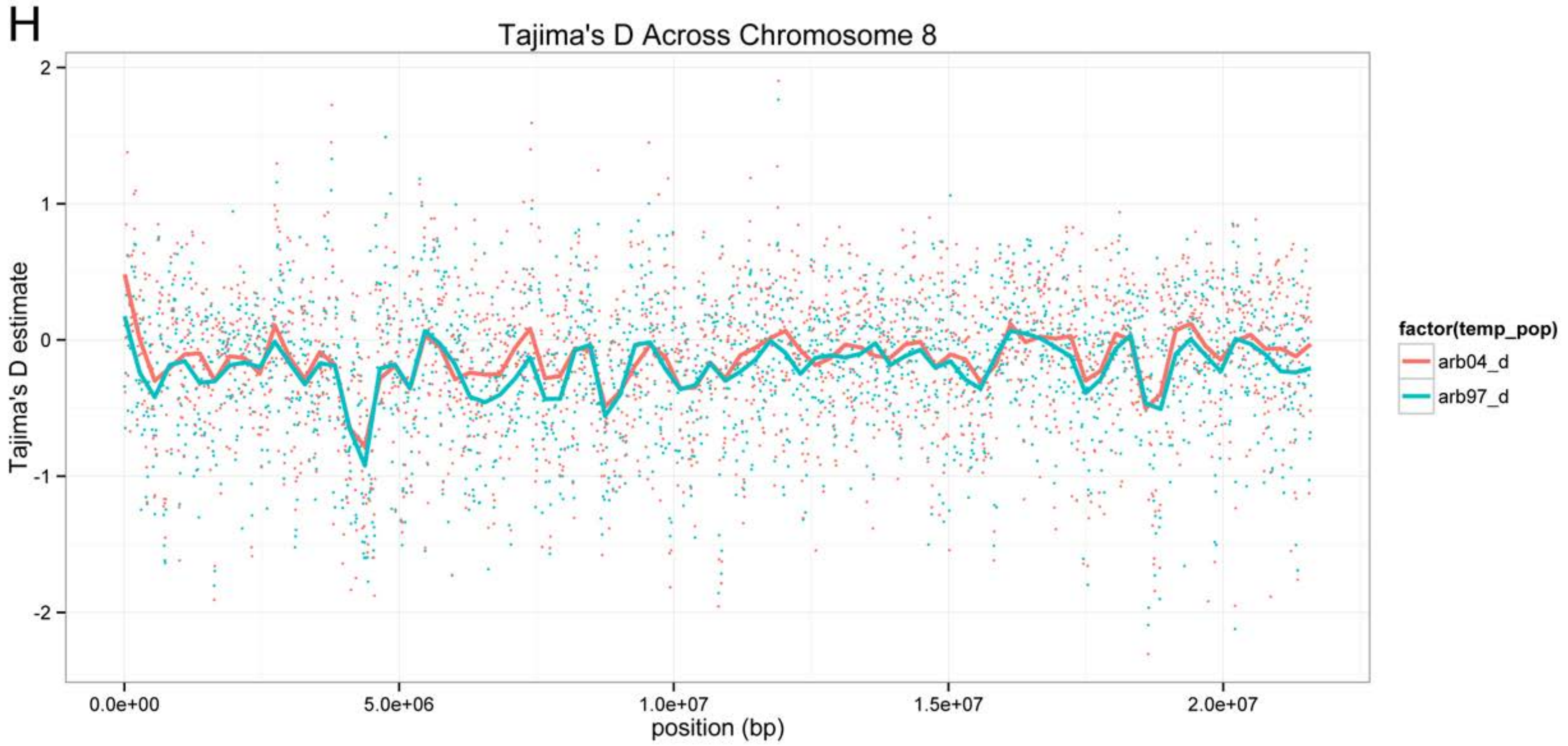
H
Tajima's D Across Chromosome 8
Tajima's D estimate
2
1
0
-1
-2
0.0e+00
5.0e+06
1.0e+07
1.5e+07
2.0e+07
position (bp)
factor(temp_pop)
arb04_d
arb97_d

I

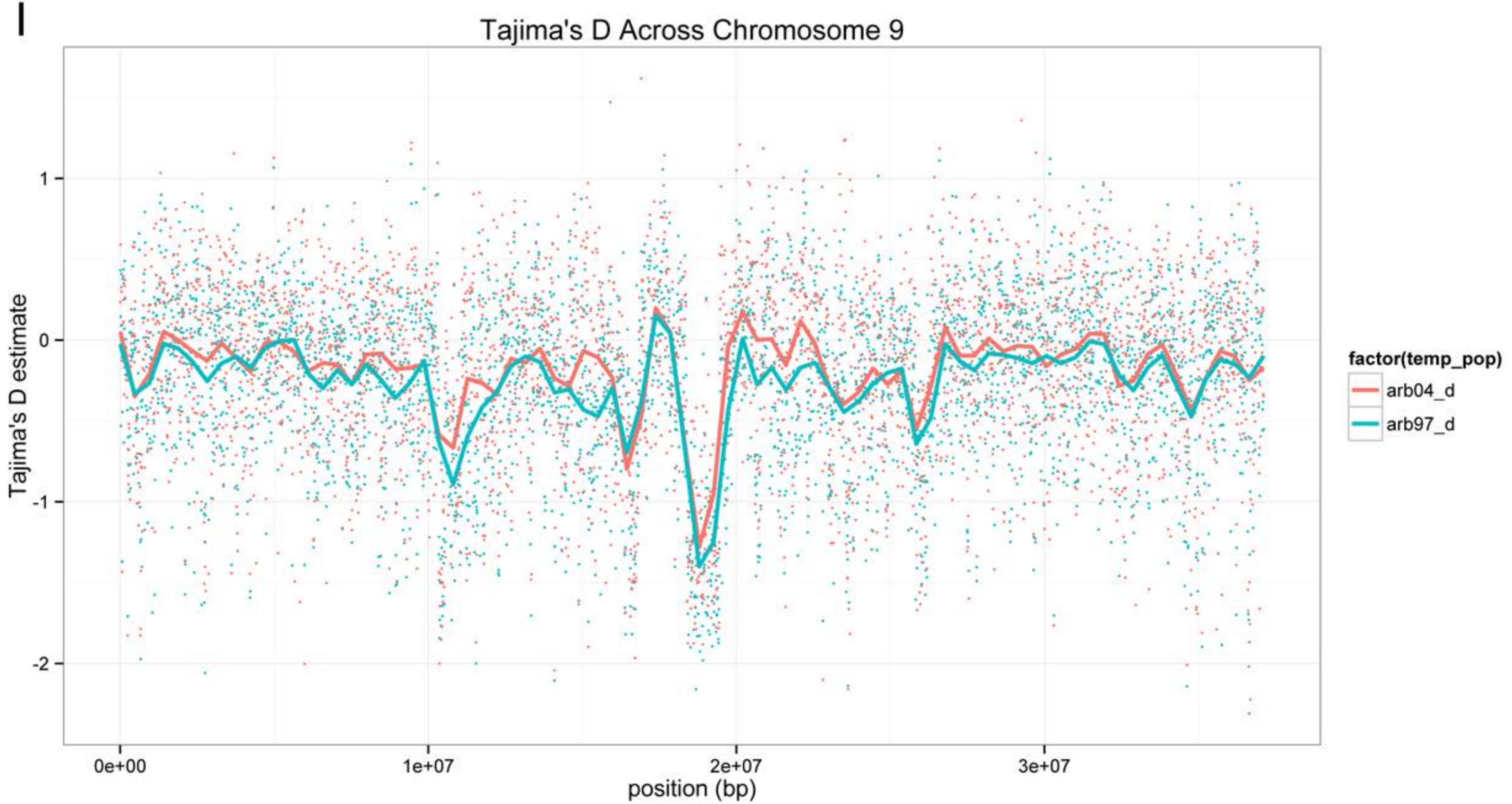
Tajima's D Across Chromosome 9
Tajima's D estimate
1
0
-1
-2
0e+00
1e+07
2e+07
3e+07
position (bp)
factor(temp_pop)
arb04_d
arb97_d

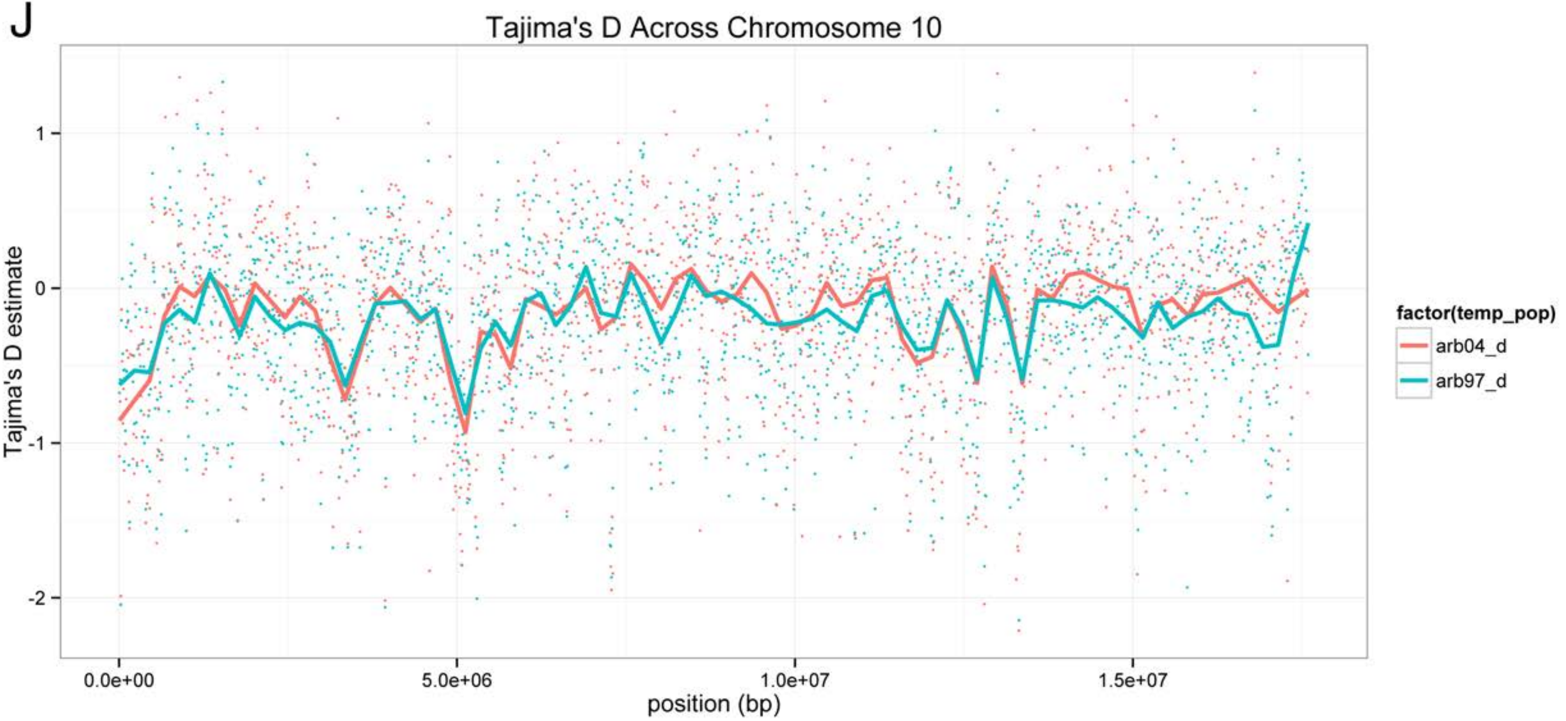
J
Tajima's D Across Chromosome 10
Tajima's D estimate
1
0
-1
-2
0.0e+00
5.0e+06
1.0e+07
1.5e+07
position (bp)
factor(temp_pop)
arb04_d
arb97_d

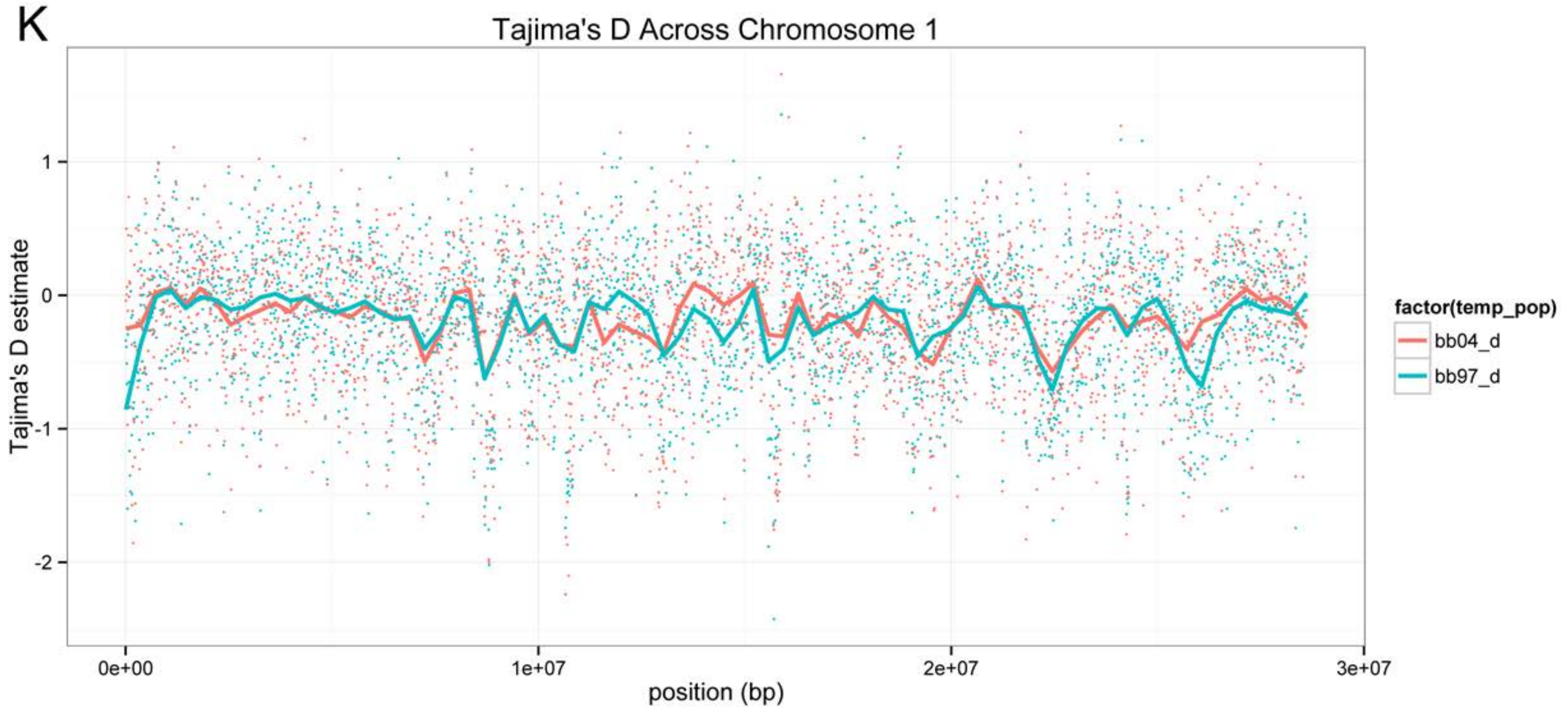

K
Tajima's D Across Chromosome 1
Tajima's D estimate
1
0
-1
-2
0e+00
1e+07
2e+07
3e+07
position (bp)
factor(temp_pop)
bb04_d
bb97_d

L

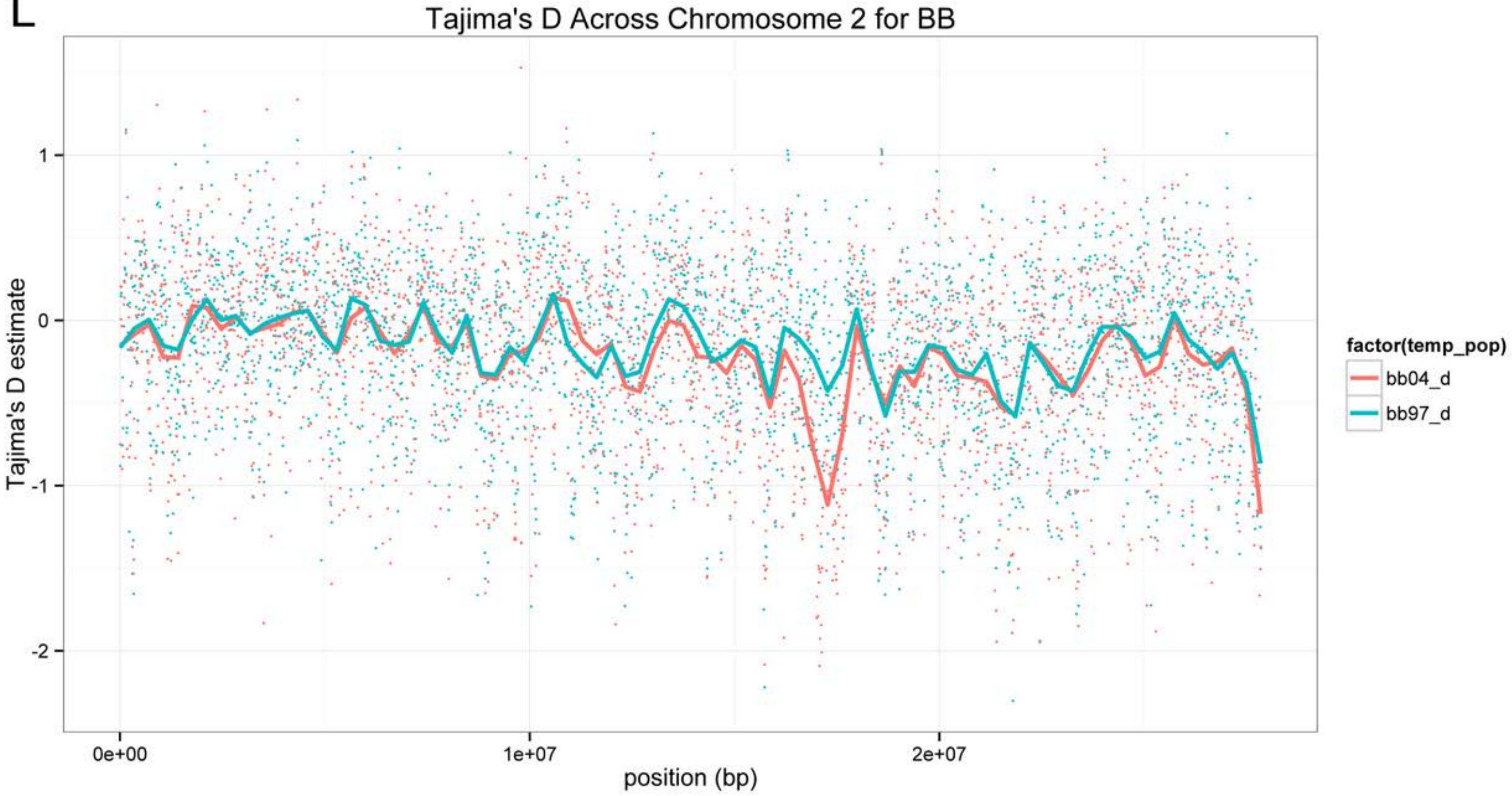
Tajima's D Across Chromosome 2 for BB
Tajima's D estimate
1
0
-1
-2
0e+00
1e+07
2e+07
position (bp)
factor(temp_pop)
bb04_d
bb97_d

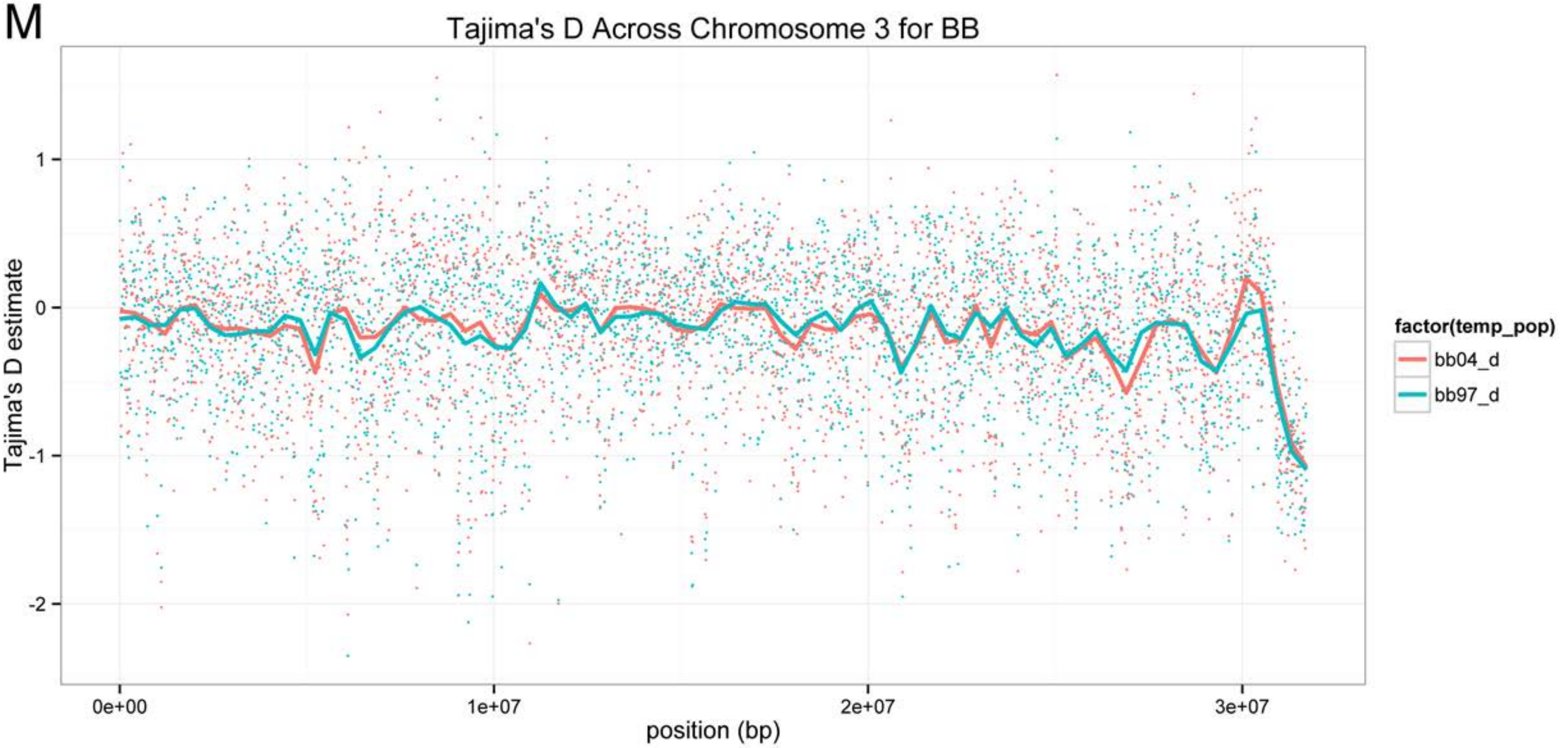
M
Tajima's D Across Chromosome 3 for BB
Tajima's D estimate
1
0
-1
-2
0e+00
1e+07
2e+07
3e+07
position (bp)
factor(temp_pop)
bb04_d
bb97_d

N

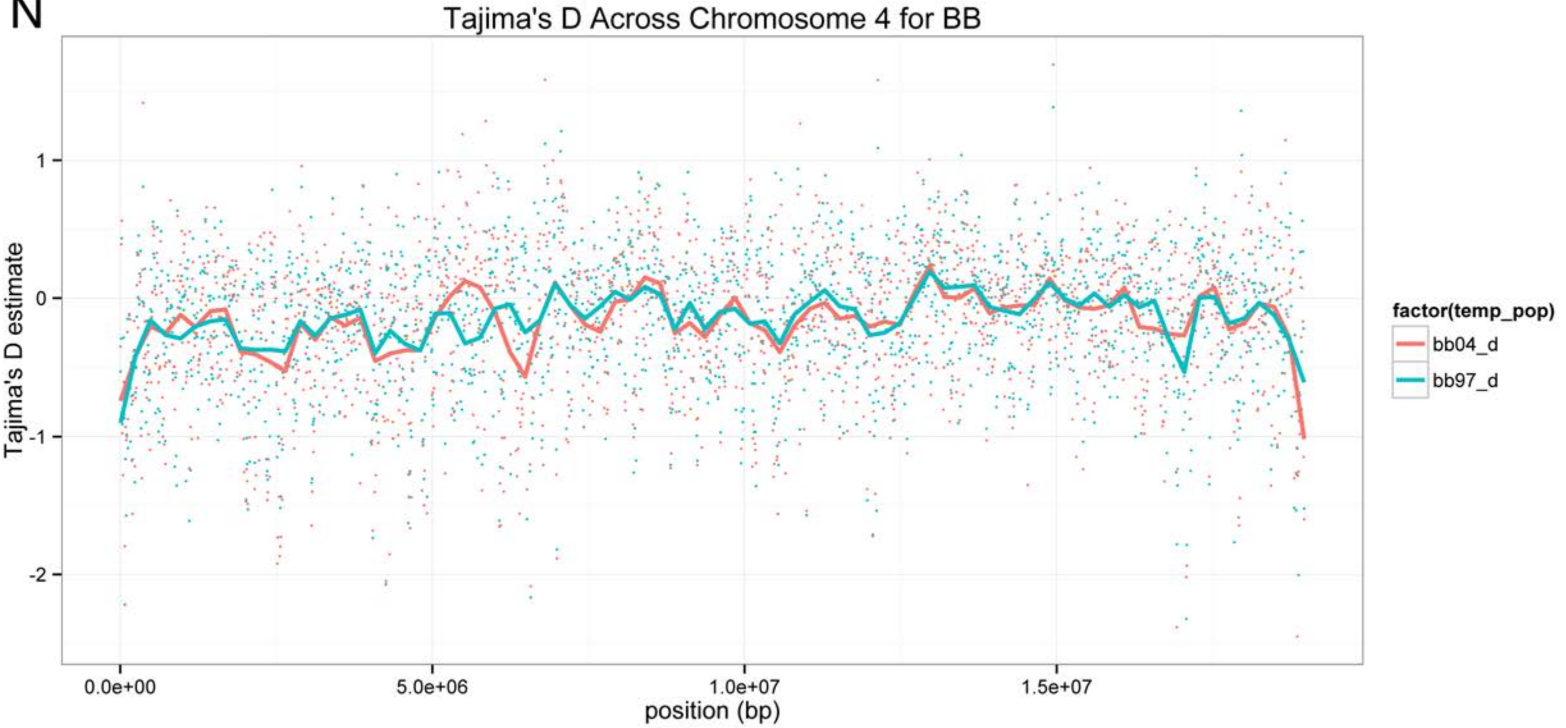

Tajima's D Across Chromosome 4 for BB
Tajima's D estimate
1
0
-1
-2
0.0e+00
5.0e+06
1.0e+07
1.5e+07
position (bp)
factor(temp_pop)
bb04_d
bb97_d

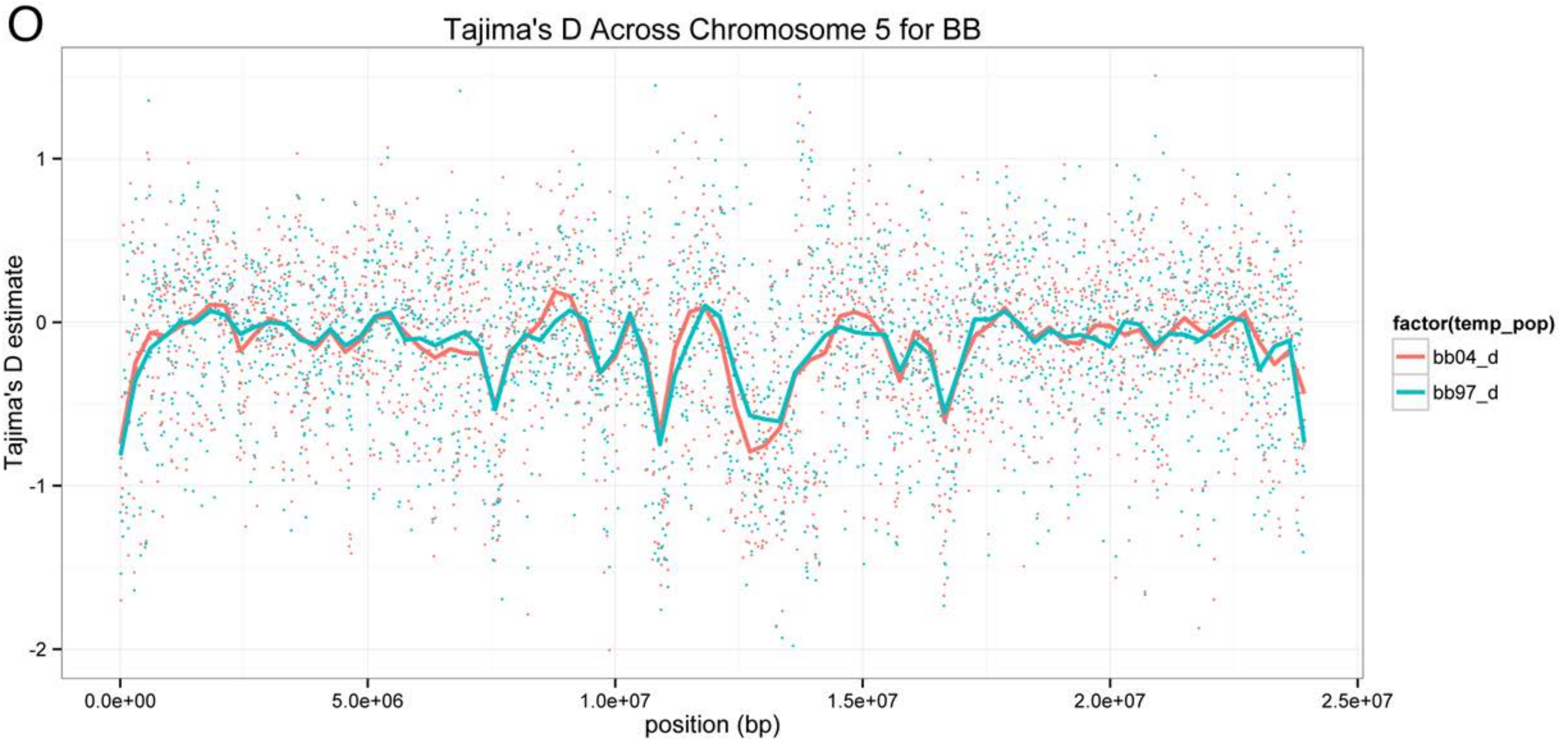

O
Tajima's D Across Chromosome 5 for BB
Tajima's D estimate
1
0
-1
-2
0.0e+00
5.0e+06
1.0e+07
1.5e+07
2.0e+07
2.5e+07
position (bp)
factor(temp_pop)
bb04_d
bb97_d

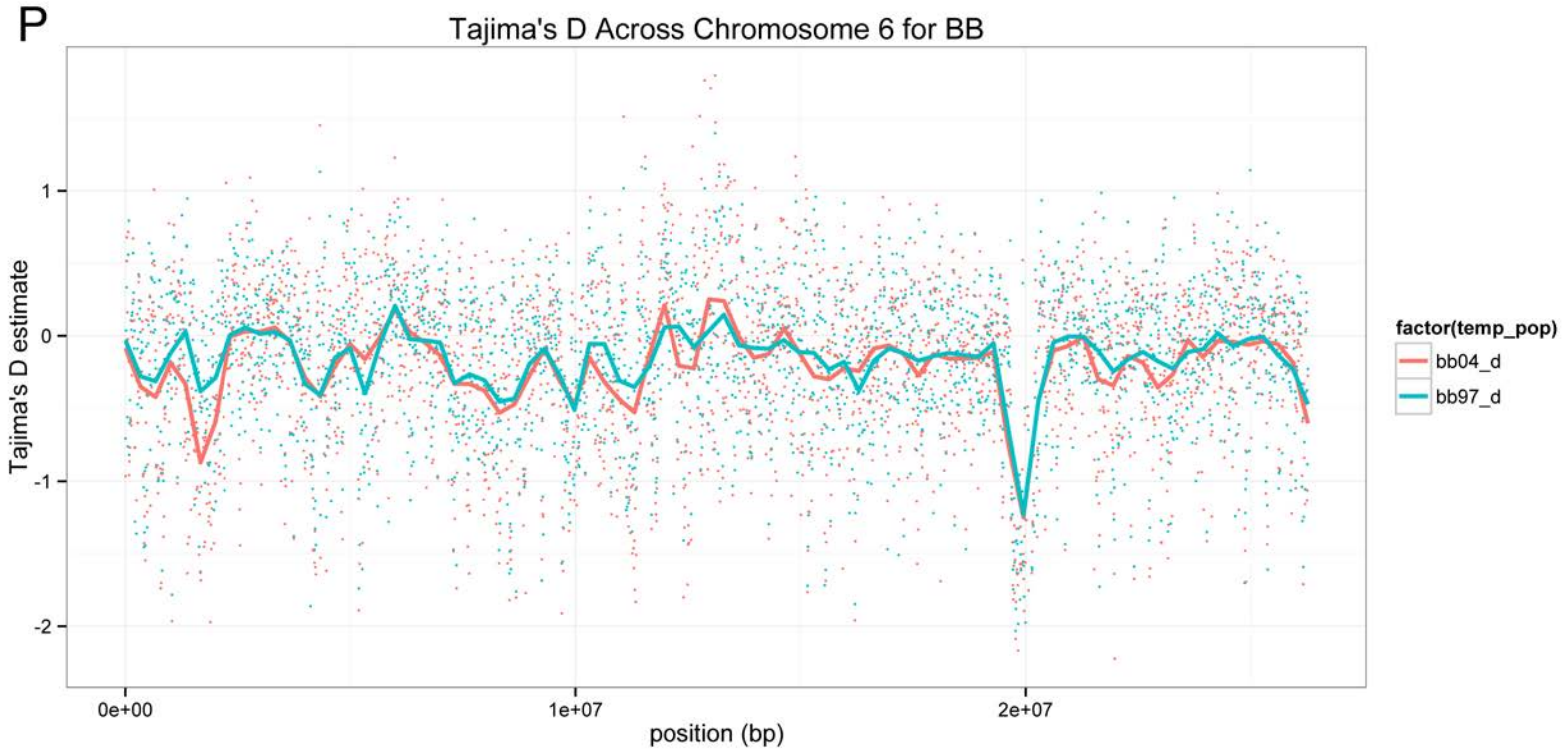
P
Tajima's D Across Chromosome 6 for BB
Tajima's D estimate
1
0
-1
-2
0e+00
1e+07
2e+07
position (bp)
factor(temp_pop)
bb04_d
bb97_d

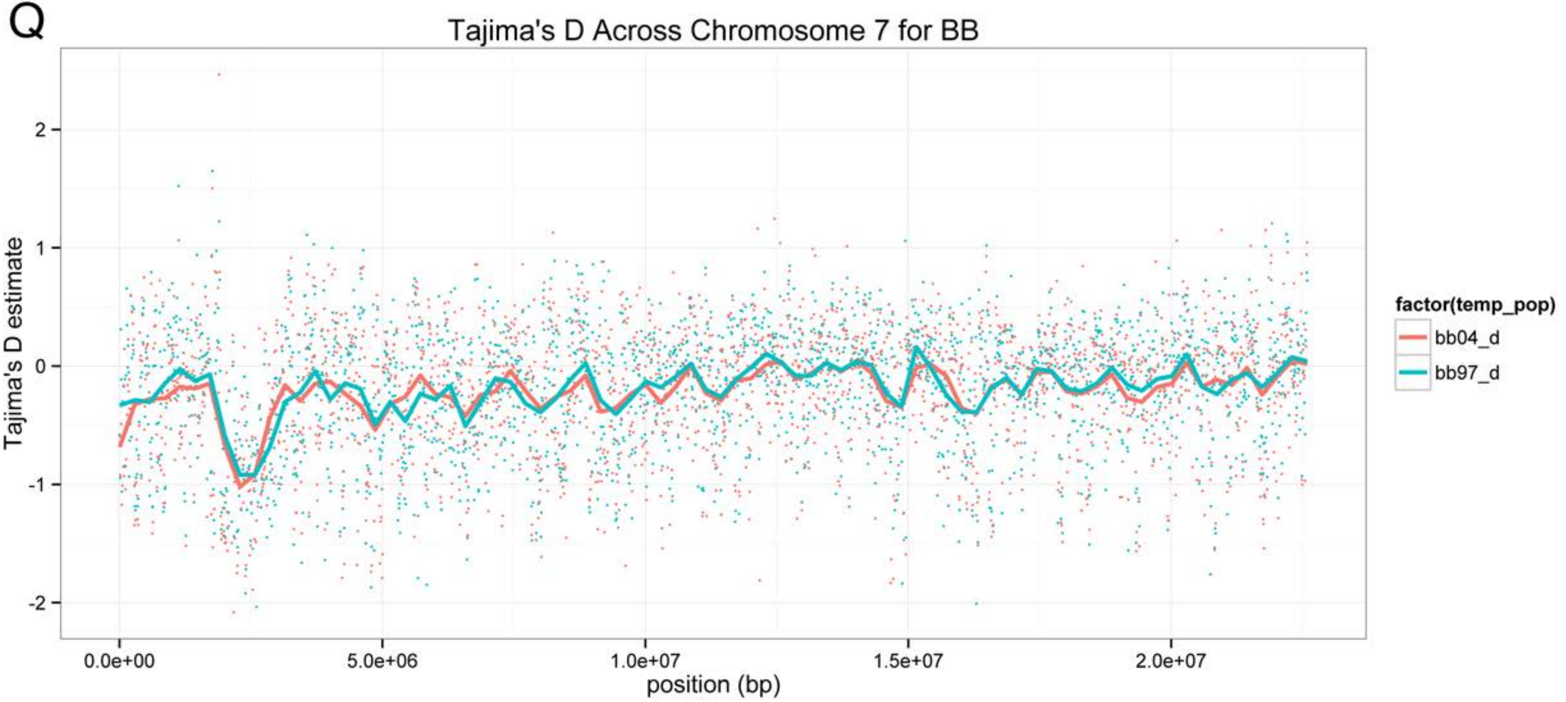
Q
Tajima's D Across Chromosome 7 for BB
Tajima's D estimate
2
1
0
-1
-2
0.0e+00
5.0e+06
1.0e+07
1.5e+07
2.0e+07
position (bp)
factor(temp_pop)
bb04_d
bb97_d

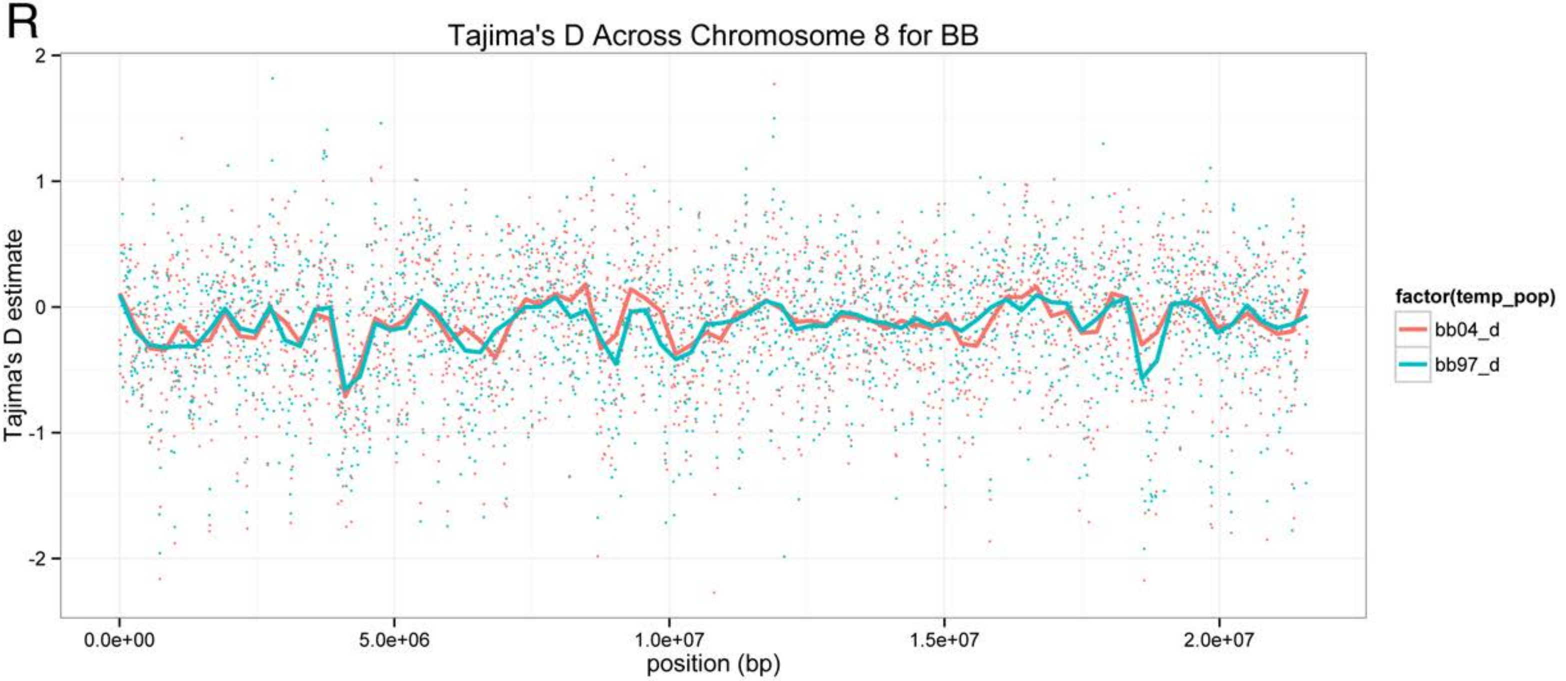
R
Tajima's D Across Chromosome 8 for BB
Tajima's D estimate
2
1
0
-1
-2
0.0e+00
5.0e+06
1.0e+07
1.5e+07
2.0e+07
position (bp)
factor(temp_pop)
bb04_d
bb97_d

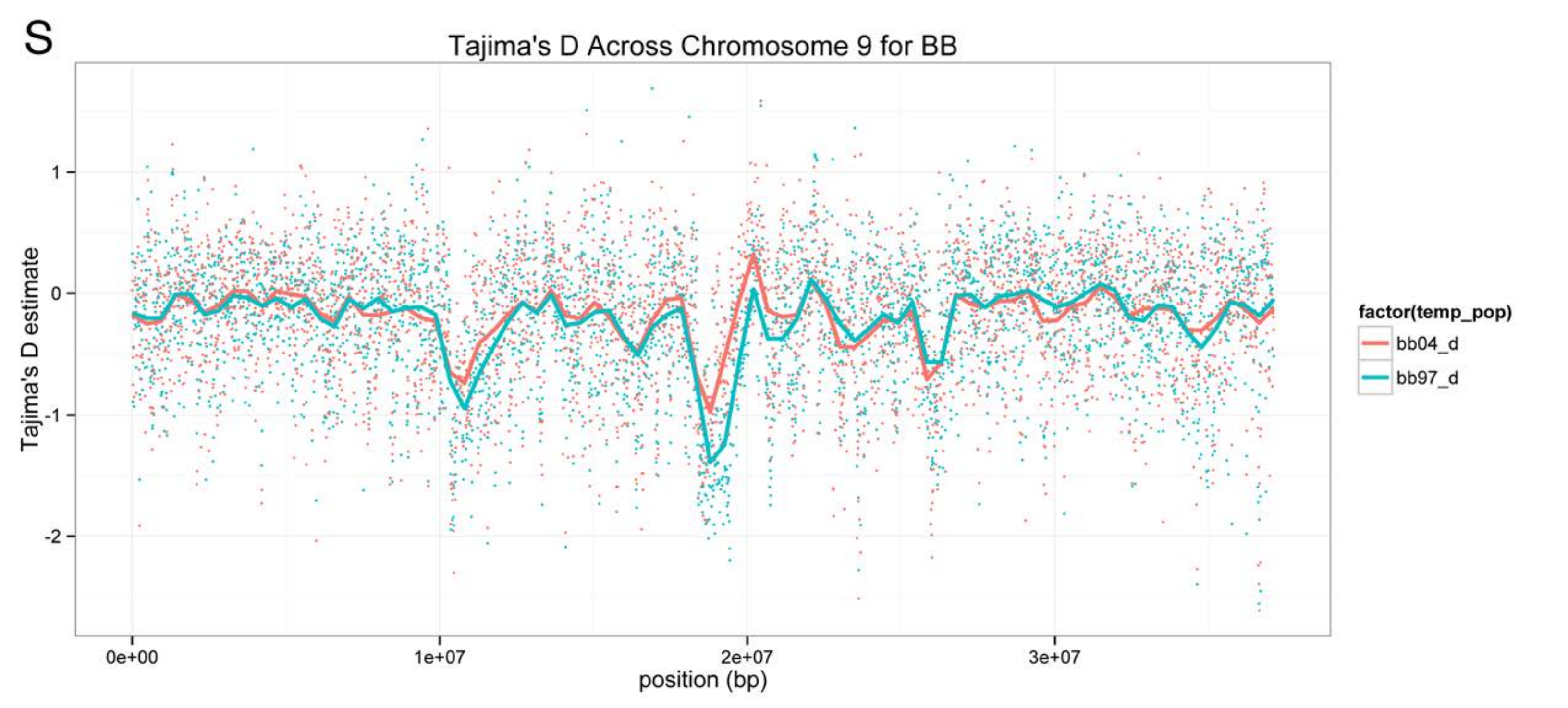
S
Tajima's D Across Chromosome 9 for BB
Tajima's D estimate
position (bp)
0e+00
1e+07
2e+07
3e+07
1
0
-1
-2
factor(temp_pop)
bb04_d
bb97_d

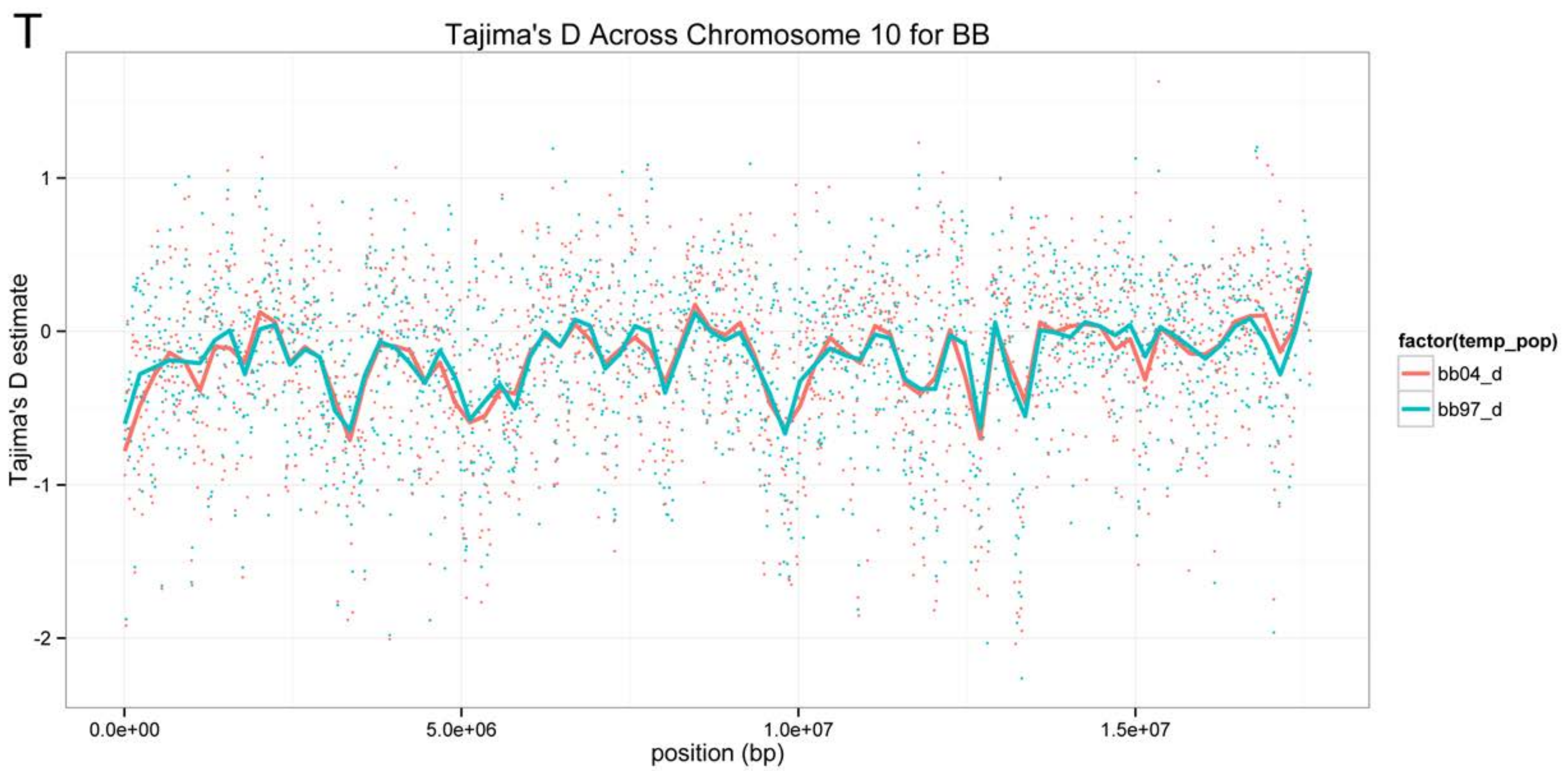
T
Tajima's D Across Chromosome 10 for BB
Tajima's D estimate
1
0
-1
-2
0.0e+00
5.0e+06
1.0e+07
1.5e+07
position (bp)
factor(temp_pop)
bb04_d
bb97_d
