# Supplementary material for: Rapid genome‐wide evolution in Brassica rapa populations following drought revealed by sequencing of ancestral and descendant gene pools
Source: Mol Ecol. 2016 Apr 13;25(15):3622–31. doi: 10.1111/mec.13615 (PMC4963267; doi:10.1111/mec.13615)
Supplement: Supplementary file 3 — Table S1. SNPs chosen for KASP validation of Brassica rapa samples. [file MEC-25-3622-s003.docx]

**Table S1.** SNPs chosen for KASP validation of *Brassica rapa* samples. ‘Chr’ stands for chromosome, NS stands for nonsynonymous, and Syn. stands for synonymous. Letters in the SNP Effect column are standard single letter amino acid codes, and letters in the SNP column are IUPAC nucleotide ambiguity codes.

| **Gene** | **Gene Description** | **Chr** | **SNP Location** | **SNP Effect** | **SNP** |
| --- | --- | --- | --- | --- | --- |
| Bra022192 | Phytochrome B | 5 | 19188889 | NS Q-K | K |
| Bra035723 | FRIGIDA | 10 | 12527985 | NS A-P | S |
| Bra009897 | GRAS family transcription factor | 6 | 17965491 | NS. L-I | M |
| Bra026583 | Pectate lyase | 2 | 20419294 | Syn. | Y |
| Bra036875 | Receptor like protein 47 | 1 | 12478011 | NS N-D | Y |
| Bra023306 | NADPH:quinone oxidoreductase | 9 | 19688761 | NS Q-E | S |
| Bra037904 | Unknown protein | 9 | 11464309 | Syn. | K |
| Bra026212 | Ankyrin repeat family protein | 6 | 5560201 | Syn. | R |
| Bra008462 | 5’ UTR BRIZ2 (BRAP2 RING ZnF UBP domain-containing protein) | 2 | 15792573 | - | M |
| *Bra000759 | UTR Disease resistance protein | 3 | 12915914 | - | S |

*Bra000759 failed to amplify for most samples, and was therefore excluded from further analysis.
