# Supplementary material for: Rapid genome‐wide evolution in Brassica rapa populations following drought revealed by sequencing of ancestral and descendant gene pools
Source: Mol Ecol. 2016 Apr 13;25(15):3622–31. doi: 10.1111/mec.13615 (PMC4963267; doi:10.1111/mec.13615)
Supplement: Supplementary file 4 — Table S2. Genes showing evolutionary shifts in both populations. [file MEC-25-3622-s004.doc]

**Table S2.** Genes showing evolutionary shifts in both populations.

| **Brassica gene** | **Chr.** | **Pos. (Mb)** | **F_ST_ Arb** | **F_ST_ BB** | **Arabidopsis homologue** | **gene name** | **gene annotation** | **GO categories** |
| --- | --- | --- | --- | --- | --- | --- | --- | --- |
| Bra023776 | 1 | 19.9 | 0.20 | 0.15 | AT3G04620 |  | Alba DNA/RNA-binding protein | Other cellular processes; response to stress; other metabolic processes; response to abiotic or biotic stimulus; developmental processes; DNA or RNA metabolism |
| Bra038723 | 1 | 26.4 | 0.21 | 0.21 | AT3G12410 |  | Polynucleotidyl transferase, ribonuclease H-like superfamily protein |  |
| Bra008462 | 2 | 15.8 | 0.22 | 0.29 | AT2G26000 | BRIZ2 | zinc finger (C3HC4-type RING finger) family protein | Other cellular processes; other metabolic processes; developmental processes; protein metabolism |
| Bra000731 | 3 | 12.7 | 0.17 | 0.24 | AT4G11040 |  | Protein phosphatase 2C family protein | developmental processes |
| Bra018326 | 5 | 7.5 | 0.20 | 0.17 | AT2G30230 |  |  | unknown biological processes |
| Bra019917 | 6 | 3.7 | 0.25 | 0.19 | AT2G39540 |  | Gibberellin-regulated family protein | Other cellular processes; other biological processes; signal transduction |
| Bra038400 | 9 | 10.9 | 0.19 | 0.18 | AT3G14940 | ATPPC3,PPC3 | phosphoenolpyruvate carboxylase 3 | Other cellular processes; response to stress; other metabolic processes; other biological processes; transport; electron transport or energy pathways |
| Bra023306 | 9 | 19.7 | 0.32 | 0.17 | AT3G27890 | NQR | NADPH:quinone oxidoreductase | Other cellular processes; response to stress; other metabolic processes; response to abiotic or biotic stimulus; other biological processes; signal transduction; transport; electron transport or energy pathways; cell organization and biogenesis |
| Bra023294 | 9 | 19.8 | 0.17 | 0.17 | AT1G32920 |  |  | Other cellular processes; response to stress; other metabolic processes; response to abiotic or biotic stimulus; other biological processes; signal transduction |
| Bra033326 | 10 | 4.3 | 0.24 | 0.14 | AT1G45063 |  | copper ion binding;electron carriers |  |
| Bra009135 | 10 | 15.4 | 0.15 | 0.16 | AT5G05800 |  |  | response to abiotic or biotic stimulus; developmental processes; unknown biological processes |
