# Supplementary material for: Rapid genome‐wide evolution in Brassica rapa populations following drought revealed by sequencing of ancestral and descendant gene pools
Source: Mol Ecol. 2016 Apr 13;25(15):3622–31. doi: 10.1111/mec.13615 (PMC4963267; doi:10.1111/mec.13615)
Supplement: Supplementary file 6 — Data S2. Methods. [file MEC-25-3622-s006.docx]

**Supplementary Materials**

**Supplementary Methods**

Technical Replicates:

The two technical replicates were combined for each pool. Technical replicates had an average F_ST_ < 0.001, indicating little difference due to sampling, with differences between populations significantly higher than between replicates (p < 0.0001). F_ST_ values between populations using different replicates were highly correlated (Pearson’s product moment correlation = 0.77).

Population genetic statistics:

The parameters are based on ([Pandey *et al.* 2011](#_ENREF_3)). We chose coverage and window size values based on Popoolation guidelines which they have demonstrated to produce highly reliable estimates ([Kofler *et al.* 2011](#_ENREF_2)). Tajima’s D was calculated using the *Variance-sliding.pl* script from Popoolation ([Pandey *et al.* 2011](#_ENREF_3)) with changes to the default parameters noted:

| Parameter | Value | Explanation |
| --- | --- | --- |
| window-size | 100000 | Length of window in bp used in calculating estimate. This size was chosen to include enough SNPs and based on Popoolation guidelines |
| step-size | 10000 | Length in bp of overlap between adjacent windows used in calculating estimate. This size was chosen to include enough SNPs and based on Popoolation guidelines |
| min-count | 2 | The minimum number of times that an allele for an identified SNP must occur. Default size was used. |
| min-qual | 20 | Only windows with a minimum quality score of 20 were included in the analysis to ensure that we were not including sequencing errors. Default cutoff was used. |
| pool-size | 50 | Number of individuals in the pool. Popoolation does not allow unequal pool sizes. This number was calculated based on our average number of pooled individuals in our 8 pools. |
| min-coverage | 8 | Only windows with this minimum coverage were included in the analysis to ensure that we were only analyzing mapped regions. Default was 4 but we increased to be more stringent. |
| max-coverage | 60 | Only windows with this maximum coverage were included in the analysis to ensure that we were not including misaligned duplicate genes. Default value is 300 but we lowered (made more stringent) as recommended by Popoolation because our study species has many duplicate genes. |

Pairwise F_ST_ calculated using the *fst-sliding.pl* script from Popoolation ([Pandey *et al.* 2011](#_ENREF_3)) with the following changes to the default parameters:

| Parameter | Value | Explanation |
| --- | --- | --- |
| min-count | 2 | The minimum number of times that an allele for an identified SNP must occur. Default size was used. |
| min-coverage | 8 | Only windows with this minimum coverage were included in the analysis to ensure that we were only analyzing mapped regions. Default was 4 but we increased to be more stringent. |
| max-coverage | 60 | Only windows with this maximum coverage were included in the analysis to ensure that we were not including misaligned duplicate genes. Default value is 300 but we lowered (made more stringent) as recommended by Popoolation because our study species has many duplicate genes. |
| min-covered-fraction | 0.0 | Fraction of window having minimum coverage. |
| pool-size | 50 | Number of individuals in the pool. Popoolation does not allow unequal pool sizes. This number was calculated based on our average number of pooled individuals in our 8 pools. |
| window-size | 100000 | Length of window in bp used in calculating estimate. This size was chosen to include enough SNPs and based on Popoolation guidelines |
| step-size | 10000 | Length in bp of overlap between adjacent windows used in calculating estimate. This size was chosen to include enough SNPs and based on Popoolation guidelines |

**Dataset S1.** All F_ST_ outlier genes, which evolved during the course of a drought. See text file uploaded separately. All outlier genes for Arboretum (Arb) or Back Bay (BB) populations including *B. rapa* gene ID, chromosome location (Chr), position in genome (position_Mb), F_ST_ for each population Arb and BB, Arabidopsis homologue, gene name, and gene annotation.

**Table S1.** SNPs chosen for KASP validation of *Brassica rapa* samples. ‘Chr’ stands for chromosome, NS stands for nonsynonymous, and Syn. stands for synonymous. Letters in the SNP Effect column are standard single letter amino acid codes, and letters in the SNP column are IUPAC nucleotide ambiguity codes.

| **Gene** | **Gene Description** | **Chr** | **SNP Location** | **SNP Effect** | **SNP** |
| --- | --- | --- | --- | --- | --- |
| Bra022192 | Phytochrome B | 2 | 19188889 | NS Q-K | K |
| Bra035723 | FRIGIDA | 4 | 12527985 | NS A-P | S |
| Bra009897 | GRAS family transcription factor | 6 | 17965491 | NS. L-I | M |
| Bra026583 | Pectate lyase | 2 | 20419294 | Syn. | Y |
| Bra036875 | Receptor like protein 47 | 9 | 12478011 | NS N-D | Y |
| Bra023306 | NADPH:quinone oxidoreductase | 9 | 19688761 | NS Q-E | S |
| Bra037904 | Unknown protein | 9 | 11464309 | Syn. | K |
| Bra026212 | Ankyrin repeat family protein | 6 | 5560201 | Syn. | R |
| Bra008462 | 5’ UTR BRIZ2 (BRAP2 RING ZnF UBP domain-containing protein) | 2 | 15792573 | - | M |
| *Bra000759 | UTR Disease resistance protein | 3 | 12915914 | - | S |

*Bra000759 failed to amplify for most samples, and was therefore excluded from further analysis.

**Table S2.** Genes showing evolutionary shifts in both populations**.** See supplementary data file uploaded separately. Shown are the 11 genes that were F_ST_ outliers between ancestors and descendants for both Arboretum (Arb) and Back Bay (BB) populations (see Fig. 2). GO categories identified using TAIR GO tool ([Berardini *et al.* 2004](#_ENREF_1)). Fig. S2 shows the direction of shifts for these genes. Chr. Is chromosome number, and Pos. is nucleotide position on the chromosome. Brassica gene, Arabidopsis homologue, gene name, and gene annotation are from the BRAD database (http://brassicadb.org/brad/).

**Fig. S1.** High correlation between KASP and Illumina next-generation determined allele frequencies in *Brassica rapa* populations*.* Illumina allele frequencies were determined from each of four pools (205 individual samples total). KASP frequencies were determined from 116 of those individual samples that were individually genotyped. Two populations are shown: Arboretum (Arb; solid lines) and Back bay (BB, dashed lines). Arrows point from 1997 populations to 2004 populations. Colors denote each of the four possible bases; only biallelic loci were considered. The nine loci (SNPs) genotyped by both methods are indicated by different shapes; the correlation between the two methods for each locus is provided in the legend.

**Fig. S2.** Tajima’s D, calculated using a 100 kb sliding window, shown across the genome for ancestral (blue) and descendant (red) populations with trendlines added using a local regression smoothing with a span of 0.05. All 10 chromosomes are mapped for both Arb (A-J) and BB (K-T) populations. Regions of the genome show evidence of selective sweeps for both ancestral and descendant populations.

**References**

Berardini TZ, Mundodi S, Reiser L*, et al.* (2004) Functional annotation of the Arabidopsis genome using controlled vocabularies. *Plant Physiology* **135**, 745-755.

Kofler R, Orozco-terWengel P, De Maio N*, et al.* (2011) PoPoolation: A Toolbox for Population Genetic Analysis of Next Generation Sequencing Data from Pooled Individuals. *PLoS One* **6**, e15925.

Pandey R, Kofler R, Orozco-terWengel P, Nolte V, Schlotterer C (2011) PoPoolation DB: a user-friendly web-based database for the retrieval of natural polymorphisms in Drosophila. *Bmc Genetics* **12**, 27.
